# Latent generative landscapes as maps of functional diversity in protein sequence space

**DOI:** 10.1038/s41467-023-37958-z

**Published:** 2023-04-19

**Authors:** Cheyenne Ziegler, Jonathan Martin, Claude Sinner, Faruck Morcos

**Affiliations:** 1 Department of Biological Sciences, University of Texas at Dallas, Richardson, TX 75080 USA; 2 Department of Bioengineering, University of Texas at Dallas, Richardson, TX 75080 USA; 3 Center for Systems Biology, University of Texas at Dallas, Richardson, TX 75080 USA

**Keywords:** Machine learning, Protein design, Functional clustering, Computational biophysics

## Abstract

Variational autoencoders are unsupervised learning models with generative capabilities, when applied to protein data, they classify sequences by phylogeny and generate de novo sequences which preserve statistical properties of protein composition. While previous studies focus on clustering and generative features, here, we evaluate the underlying latent manifold in which sequence information is embedded. To investigate properties of the latent manifold, we utilize direct coupling analysis and a Potts Hamiltonian model to construct a latent generative landscape. We showcase how this landscape captures phylogenetic groupings, functional and fitness properties of several systems including Globins, *β*-lactamases, ion channels, and transcription factors. We provide support on how the landscape helps us understand the effects of sequence variability observed in experimental data and provides insights on directed and natural protein evolution. We propose that combining generative properties and functional predictive power of variational autoencoders and coevolutionary analysis could be beneficial in applications for protein engineering and design.

## Abstract (web summary)

In this work, the authors study protein families’ VAE latent manifolds and coevolutionary Hamiltonians. These Latent Generative Landscapes predict phylogenetic groupings, fitness & functional properties for several systems with clear protein engineering/design potential.

## Introduction

During the process of evolution, proteins are subject to changes in their amino acid composition via mutation, insertions, deletions, and gene duplication. These changes are constrained by fitness and selective pressures as determined by the overall structure, function, stability, and folding of protein sequences in the organism that encodes them^1,2^. These constraints impose statistical signatures in the collection of evolutionarily related sequences that allow features, such as structure, function, and interactions, to be reconstructed from homologous sequence alignments using methods such as direct coupling analysis (DCA), GREMLIN, and EVcouplings^3–6^. These methodologies offer excellent performance in identifying relevant amino acid interactions useful for structure inference^7–10^, complex formation^5,11–14^, molecular specificity^15–19^, the effects of protein mutations^20–22^, and protein design, including engineering of functional proteins with specific properties, such as repressors^23^, fluorescent proteins^24,25^, and enzymes^26^, and can be used to inform evolutionary models^27^, but they lack strong performance in classifying specific functions of a given protein. Recent focus has shifted towards using state-of-the-art machine learning approaches. Notable methods to predict protein structures include Alphafold^28^ and end-to-end differentiable learning^29^, but other machine learning models have been used to understand protein sequence attributes that are correlated with certain functions and outcomes, such as DeepPPI for interactions^30^, restricted Boltzmann machines (RBM) to detect motifs associated with function^31^, and variational autoencoders (VAE) for phylogenetic clustering and predicting effects of protein mutation^32,33^. Architectures such as VAE^34^ and Transformers^35^ are also capable of generating proteins. In this work, we address further capabilities of the VAE, an unsupervised and generative machine learning model, to study the evolution and function of protein families.

VAEs consist of an encoder and a decoder. The encoder compresses input data (**x**) into a latent variable ensemble (**z**), where **z** has been constrained into a multivariate latent distribution with an approximated Gaussian prior. The decoder then takes the encoded variable **z** and reconstructs the input data in a Bayesian framework^36,37^. The embedding of data into the lower-dimension latent manifold creates a continuous latent space that can be sampled to generate new data based on the approximated posterior distribution learned by the encoder. These samplings of the latent space from the VAE architecture can be sufficient to create new objects^38^ and are capable of generating new protein sequences when trained on a family of proteins^34,39,40^.

Since VAEs approximate a posterior distribution, they are powerful tools to cluster data in a latent space and have become a popular replacement for principal component analysis (PCA). Their performance is proven to be comparable with robust PCA models^41^ and has been employed on biological data sets with favorable results^32,42,43^. Previous analyses of protein sequences in latent space have shown the organization of sequences into phylogenetic clusters^32,44^, but the latent space embedding itself was not investigated. We propose that combining the predictive power of coevolutionary models with the classification and generative power of the VAE would better inform the analysis, generation, and modification of protein sequences in a manner that requires no labeling information. Through this combination of statistical models, we create a latent generative landscape (LGL), where accessible VAE sequence space is assessed using the inferred fitness from DCA. By exploring a large amount of sequence space, we have uncovered a new method to traverse the diversity of functional space in proteins that is more flexible than other architectures, such as transformers^45,46^ and generative adversarial networks (GANs)^47^, due to higher diversity of encoded possible sequences, no required labeling, and easily accessed latent representations. Specifically, the LGL provides a framework to rationally sample and traverse latent space where certain protein attributes may be selected without input of labeling information. We show how this LGL can be applied to multiple protein systems including the identification of functional features in the family of globins, exploring diversity in fitness in *β*-lactamases, local functional details of cold sensitive proteins, the pathways of directed evolution in transcription factors as well as the analysis of evolutionary propagation of spike proteins in coronaviruses. This framework to study the sequence space of complete protein families serves as a conceptual and quantitative map to get insights into fitness, functional diversification, and a guide for generative protein design. We also developed software for interactive visualization of these landscapes that we have made available for others to use.

## Results

### LGL captures phylogenetic, function, and fitness information

**Fig. 1:**
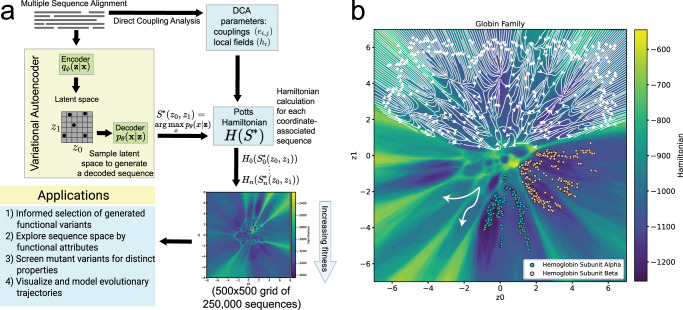
An overview of latent generative landscape (LGL) methodology. **a** A schematic overview of Hamiltonian mapping of VAE latent space and its applications. Using a multiple sequence alignment as input, DCA and VAE models are independently trained. Maximum probability grid-sampled sequences (S^*^) from the VAE latent space are then scored by DCA with a Hamiltonian value, *H*(S^*^), to create the latent generative landscape. The landscape may then be used for various applications, including de novo protein generation, protein engineering, protein classification, and evolutionary trajectories. **b** A schematic overview of the LGL. In the bottom left, an example of mutational paths on the landscape. On top, a streamplot of re-encoding vectors from generated sequences highlighting strong correspondence of the encoder-decoder relationship to the latent generative landscape. On the right, the embedding of sequences in the basins of favorable Hamiltonian values of landscape. The landscape color is defined by the DCA Hamiltonian of the maximum probability sequence generated by the decoder at that point. This sample landscape constitutes a total of 250,000 sequences.

To learn sequence attributes that confer certain properties to protein families, we compiled multiple sequence alignments (MSAs) for each family of interest using PFAM and HMMER^48,49^. The MSA is then fed into two separate models. First, the MSA is defined as a set of binary matrices where each sequence *x* is a 23 by *L* matrix. Rows encode all possible amino acid characters, including a gap character, selenocysteine, and pyrrolysine, and *L* is the length of *x*. Then, the input matrices are used as the training data set for the VAE. The VAE architecture consists of an encoder module and decoder module, which are connected using two latent variables, *z*_0_ and *z*_1_ (Fig. 1a). The encoder approximates the true posterior probability distribution *p*_*θ*_(*z*∣*x*), defined on the parameters *θ*, using a family of distributions *q*_*ϕ*_(*z*∣*x*), defined on parameters *ϕ*, which are the trainable weights of the encoder network. The learned distribution by the decoder, *p*_*θ*_(*x*∣*z*), is approximated to be a multivariate Gaussian. The latent space constructed by *z*_0_ and *z*_1_ is treated as a manifold, which can be sampled to generate new sequences. Each coordinate in a 500 × 500 grid is sampled from the latent space manifold to generate the maximum likelihood sequence at the central coordinate and is represented by a pixel in a landscape plot (Fig. 1a).

Secondly, the MSA is used to perform DCA^3^. DCA is a global statistical inference model, in which the maximum entropy principle is utilized to derive a joint probability distribution of sequences parameterized by residue position couplings (*e*_*i*,*j*_) and local fields (*h*_*i*_) representing independent statistics. These parameters may then be used as input to a large-q Potts Hamiltonian model^50–52^. By applying the Hamiltonian function to each coordinate-associated sequence in the 500^2^ pixel grid, we construct the latent generative landscape (LGL) in which sequence space is organized by the VAE latent variables and the learned distribution, *q*_*ϕ*_(*z*∣*x*), and the sequence “energy” to traverse the space is defined as the Hamiltonian, Fig. 1a. Thus, we are retrieving the VAE’s innate encoding of fitness using the Hamiltonian value as a score^50^. While the VAE’s assessment of fitness could also be estimated using the VAE’s ELBO score (Equation (4)) and the ELBO is correlated to the DCA Hamiltonian (see Supplementary Fig. 1), the Hamiltonian score has ample evidence demonstrating its utility and predictive capabilities for functional outcomes in proteins^20,26,52–54^.

As shown in Fig. 1, the VAE is subsampled in a grid-like fashion where each element of the 500^2^ pixel grid represents a generated sequence (Equation (8)) by its associated Hamiltonian score. Although different pixel-grid densities can be selected, this choice of pixel density is sufficient to capture the complexity of our latent space (see Supplementary Fig. 2). This scoring procedure uncovers an elaborate landscape of sequences where basins of low Hamiltonian sequences are surrounded by barriers of high Hamiltonian sequences, with the sequences used in the training of the VAE typically being embedded in the basins. Higher order clustering of sequences can be seen in which the barriers encapsulate smaller clusters of sequences under a single larger classification, which could provide an improvement on the unsupervised classification capabilities of the VAE model alone. Furthermore, this latent generative landscape may assist users in generating functional sequences with desired properties. Although more than two latent dimensions can be used to produce models with smaller training loss for the purpose of generating sequences^40^, we show in Supplementary Fig. 3 that, using a statistical validation outlined in the next section, two dimensions are sufficient to capture the critical statistics of protein families and adding more dimensions does not necessarily indicate an improvement, a result which is consistent with other work^55^. Given this, we expect that the LGL could be used in the functional design of proteins. Using the landscape, areas with less favorable fitness and/or lower family-likeness can be avoided to aid functional sequence generation. To generate sequences with particular attributes, users may sample within global or local basins where proteins with known properties are embedded, providing a clear subset of sequence space in which to sample. Similarly, a known protein may be mutated in silico towards a new, known function by tracking mutants’ escape from a basin and movement into sequence space associated with the new function.

Additionally, the positional relationship between encoded sequences within the latent space suggests an attractive model for studying sequence evolution and phylogenetic relationships, hearkening back to older theories of evolutionary landscapes^56^ where genes were imagined as points on landscapes of high and low fitness. One way of understanding the latent space organization is shown in the upper section of Fig. 1b with a vector plot, where at each pixel coordinate, a maximum probability sequence is generated and then re-encoded through the encoder, yielding vectors of coordinate change. Sequences generated on Hamiltonian barriers are generally re-encoded within the basins and towards what appears to be central regions of little coordinate change. This re-encoding has strong correspondence with the visualized LGL, and further justifies the use of the decoder-generated landscape to analyze novel encoded sequences, such as evolutionary trajectories created through other methods.

**Fig. 2:**
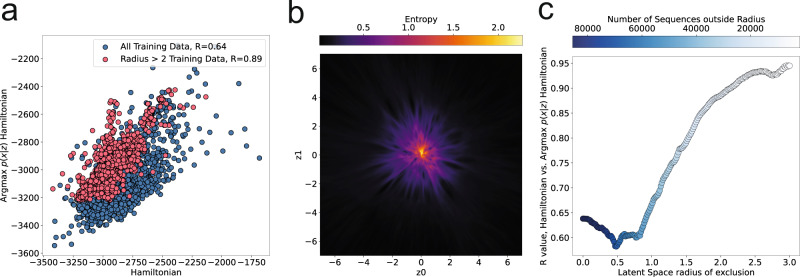
Relationship between entropy and Hamiltonian score fidelity to training sequences for the tRNA Synthetase family. **a** Comparison of Hamiltonian scores for input training sequence and $\arg \max p(x|z)$ sequence generated at training sequence’s encoded *μ* coordinate, shown at different radial exclusion distances. **b** Entropy of decoder distribution at each coordinate (Equation (11)). **c** Improvement of correlation between Hamiltonians of input and $\arg \max$ sequences through exclusion of sequences within the center. A radius is expanded where sequences lying within the radius are excluded from correlation calculation. Color is number of sequences remaining for calculation after exclusion step.

To more quantitatively measure the relationship between encoded sequences and the decoder-produced landscape, we encode training sequences into the landscape and decode through Equation (8) the maximum probability sequence at the encoded *μ* coordinate, then compare this sequence’s Hamiltonian with the input sequence’s Hamiltonian. We analyzed this relationship with the tRNA Synthetase family. Shown in blue in Fig. 2a, we find a positive correlation (*R* = 0.64) between the two values. We noticed, however, that this correlation changes depending on the variability of the decoder distributions produced by the VAE, which are higher towards the center of the plot, (Fig. 2b), and thus have higher entropy at each coordinate as calculated by Equation (11). By selectively removing sequences from our correlation calculation based on their proximity to the high entropy center, we can reliably improve the correlation of the LGL produced sequences, see Fig. 2a (red symbols, *R* = 0.89) and Fig. 2c. We note that while the correlation measured is family specific (Supplementary Fig. 4), we found, in all families tested, that correlation between the sequence Hamiltonian and LGL Hamiltonian improves notably for sequences outward from the high entropy areas.

### LGL identifies latent space for specific functions and family-likeness

**Fig. 3:**
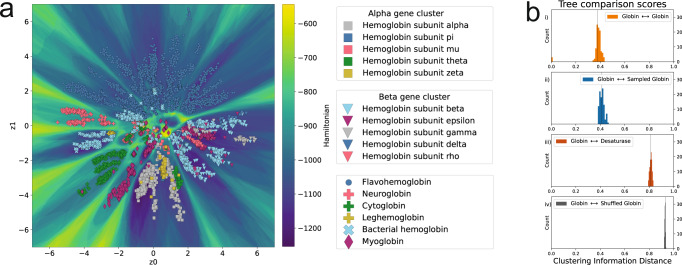
The LGL highlights phylogenetic and functional clustering. **a** An example of the global clustering shown by the Hamiltonian landscape. The alpha and beta gene cluster boxes indicate genome level clustering, labeled with squares and triangles, respectively. Other globin classifications are shown in the third legend box. **b** The VAE landscape encodes phylogenetic information. Each plot indicates 100 tree comparisons using the Clustering Information (CI) metric, comparing (i) extant sampled trees with extant sampled trees, (ii) extant sampled trees with landscape sampled trees, (iii) extant globin trees with extant desaturase trees, (iv) extant sampled trees and its leaf shuffled copy.

Across many training sets, we find that the VAE clusters sequences having members with similar phylogenetic relationships, which had been observed previously^32^. While phylogenetic information can be reconstructed from VAE models, functional information has also been shown to be encoded^33^. Using the latent generative landscape, we show how the underlying landscape encodes phylogenetic and functional information. In the LGL for Globin family (PF00042)^48^, clusters are defined by functional classification, where individual clusters have sequences with similar classifications from Uniprot, Fig. 3a. The alpha and beta hemoglobin clusters are indicated because they each contain sets of genes that exist in the same locus on different genomes, with different regulatory mechanisms coordinating their differential expression during the development of many vertebrate organisms^57^. The high Hamiltonian barrier separating these two clusters, which is not present between the clusters within the bubble, allows additional classification power to distinguish between these two coevolving sets of sequences.

Additionally, the training set for this model included many sequences which either lacked clear annotation or had more obscure classifications. Many of these sequences could be better annotated based on the most common constituents of the basins they exist in, as shown in Supplementary Fig. 5. While other VAEs have been used to identify related sequences^44^, the LGL provides a clear subdivision of latent space rather than relying only on clusters of input sequences. The preservation of phylogenetic relationships between points is captured by the underlying landscape and can be demonstrated by comparing phylogenetic trees built from natural sequences with those built using VAE generated sequences. We used the Clustering Information (CI) metric^58^ to measure the similarity between landscape sampled trees and real sequence trees based on their topological organization (see Methods and Supplementary Fig. 6 for more details), with a score of zero meaning identical trees and a score of one indicating completely dissimilar trees (expected score for comparing random trees of this size shown in Fig. 3b(iv)). In Fig. 3b(i), pairs of trees randomly created from the same pool of real Globin sequences average a CI score of 0.36, while comparing real Globin trees to landscape sampled trees gives an average CI score of 0.41 (Fig. 3b(ii)). When Globin trees are compared with a distinct protein family, e.g., FA_Desaturase family trees (Fig. 3b(iii)), the average CI score is 0.81, indicating that the landscape generated sequences contain much of the precise sequence information to create the real Globin trees and that this information is specific to the Globin family of sequences. In this way, the latent landscape can be used as a generative phylogenetic tree, where specific phylogenetic properties can be captured and studied by sampling from basins.

**Fig. 4:**
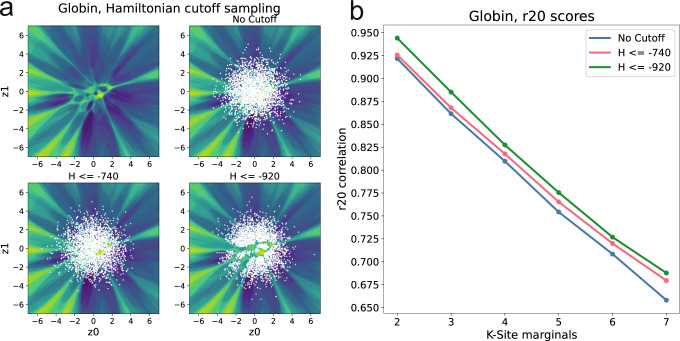
The barriers are regions of poor fit to training set statistics. **a** Sampling was performed using an ${{{{{{{\mathcal{N}}}}}}}}(0,2{{{{{{{\bf{I}}}}}}}})$ distribution, only permitting coordinates which lie below the specified Hamiltonian cutoff. A total of 10,000 sample coordinates were generated in each case, and sequences were generated through evaluating the decoded probability distribution at each point. **b** The corresponding *r20* correlation scores. Low Hamiltonian regions have better K-site marginal correlations, indicating greater statistical accuracy to the input training set.

While phylogenetic relationships between sequences are maintained within the basins, the extreme barriers between the basins indicate dramatic shifts in the output probability distributions of the decoder as distinct regions of the latent space are interpolated. One interpretation of this landscape is that the sample space of likely functional sequences in the VAE is not uniform across the Gaussian distribution prior that the decoder was trained with. We assess the statistical fidelity of the landscape through the *r20* metric^55^, computing higher order marginal statistics of VAE generated sequences and comparing them to a reference set. We compare our generated sequences to the input training set and see that by avoiding high Hamiltonian barrier regions when generating sequences we produce data sets of higher accuracy to the training data (Fig. 4). It also implies that some paths of interpolation can break amino acid couplings within a protein sequence, while others preserve these important relationships.

### Local LGL encodes important information for function and fitness

**Fig. 5:**
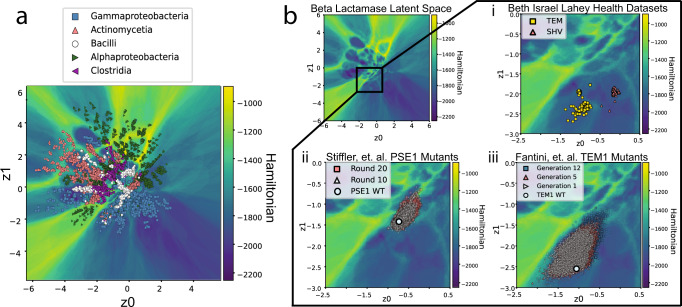
Analysis of LGL for Class-A *β*-lactamases. **a** *β*-lactamase latent space shows separation of proteins by phylogenetic information. The top 5 classes with the largest number of sequences in training MSA are shown. **b** Local LGL for a subset of class-A *β*-lactamases is expanded. (i) A local separation between TEM and SHV *β*-lactamases is observed, indicating that the LGL allows subsets of sequences with specific properties to be identified. In this case, we observe the separation of the poor oxyimino-*β*-lactam hydrolyzer: TEM, and the efficient oxyimino-*β*-lactam hydrolyzer: SHV, by a low barrier of unfavorable fitness. Determining the classification between SHV and TEM usually must be done experimentally, but with the LGL, division between these two groups based on sequence information is easily identified. (ii) Stiffler, et al.^67^ PSE-1 mutants were generated using experimental evolution, where mutants are generated using error-prone polymerase chain reaction (epPCR), screened using 6*μ*g/mL of ampicillin, and selected for survival. Subsequent rounds are generated from surviving mutants. We observe escape from the local fitness basin due to lower selection pressure and are overall more diverse than the Fantini, et al. mutants^68^. (iii) Fantini, et al. mutants were also generated using directed evolution, and we observe that mutants remain in local fitness basin due to increased selection pressure.

While the VAE is capable of clustering proteins by phylogenetic information^32,59^, we demonstrate that local differences in VAE latent space can be retrieved and interpreted using the LGL. In Fig. 5a, we analyze how the LGL of class-A *β*-lactamases (PF13354) encodes function. *β*-lactamases are a family of enzymes that hydrolyze the *β*-lactam ring of *β*-lactam antibiotics, conferring antibiotic resistance. Within the *β*-lactamase family are different classes of *β*-lactamases, which have differing mechanisms of hydrolysis^60^. Underneath class-A is a subclassification (TEM, SHV, CTX-M, KPC, CARB) that aims to define which antibiotics can be hydrolyzed by the enzyme, but these subclassifications are often difficult to define^60^. Being able to computationally define separate subclassifications and identify sequence attributes leading to overlap may assist biomedical research in identifying which antibiotics are susceptible to hydrolysis for uncharacterized *β*-lactamase variants. When taking into consideration local regions of the latent generative landscape, separations between different local clusters may become apparent, as observed with the separation between TEM and SHV *β*-lactamases, Fig. 5b(i)^61^. This shows that even less prominent basins in the LGL may represent real attributes about proteins and their evolution.

Typically, TEM and SHV classifications must be determined experimentally by the enzyme’s ability to hydrolyze oxyimino *β*-lactams, but using the LGL, we are able to subdivide sequence space into TEM and SHV subsets^62–64^. Further development of these capabilities could yield reliable prediction of antibiotic susceptibility using sequence alone, where sequences that occupy barriers are not as “fit” as the wild-type sequences but may still be functional. Furthermore, being able to predict when a bacterial population is more liable to generate new extended-spectrum *β*-lactamases (ESBLs) could help identify meaningful antibiotic rotation regimes^65,66^. To analyze movement of variants within the LGL, we consider PSE-1 and TEM-1 *β*-lactamase mutants generated by Stiffler, et al.^67^ and Fantini, et al.^68^, respectively. These experiments perform experimental evolution on class A *β*-lactamases in an attempt to create input data for structural prediction and residue contact map creation. These data sets have been used to construct contact maps and are useful in showing movement of functional, but mutating, sequences within latent space because it is assumed that important couplings and local fields are preserved. We observe the behavior of generated mutants when placed in the latent space by the encoder, Fig. 5b(ii–iii). For Stiffler, et al. PSE-1 mutants shift away from wild-type PSE-1 and the local PSE basin, crossing multiple areas of lower fitness. For Fantini, et al. TEM-1 mutants, mutants move further into the local TEM basin where the inferred fitness is more favorable, with later rounds exhibiting mild population of the CARB basin. For each set of mutants, the Δ Hamiltonian quantifying the difference between wild-type *β*-lactamase and each mutant was calculated using Equation (10). The distribution of Δ Hamiltonian scores further supports the movement toward more favorable fitness sequence space for Fantini mutants and toward less favorable or neutral fitness sequence space for Stiffler mutants (Supplementary Fig. 7). Additionally, Stiffler mutants were shown to be more diverse than Fantini mutants^54^. This difference in mutant library diversity has been attributed to differences in selection pressure between the two experiments. Stiffler mutants underwent lower selection pressure while Fantini mutants underwent a higher selection pressure^54^. While Fantini mutants appear to occupy a larger space, it is important to recall that the latent generative landscape is non-Euclidean and sequence space closer to the origin tends to encode a higher density of unique sequences (Supplementary Fig. 8). Additionally, sequence identity between mutants and their respective wild-type protein are high for both TEM1 and PSE1 mutants (Supplementary Fig. 9). This demonstrates how the LGL is capturing information beyond sequence identity. Since all sequences provided by Stiffler and Fantini are functionally able to hydrolyze *β*-lactam rings, it is important to note that unfavorable fitness does not always mean complete loss of function. The movement and diversity of Stiffler mutants, when compared to Fantini mutants, demonstrates how variants can cross Hamiltonian barriers under lowered selection pressure to access new basins of favorable fitness sequence space. Population of less favorable LGL sequence space under lowered selection was also observed for VIM-2 metallo-*β*-lactamase under treatment with varying concentrations of ampicillin, shown in Supplementary Fig. 7d. This indicates that the latent generative landscape may be useful in describing diversity of populations and movement to or away from other areas of known function. While some functional space may appear to occupy discrete regions in the LGL, it is also possible that these functions exist on a spectrum, as observed with functional *β*-lactamase sequences occupying LGL barriers. Sequences encoded in barriers should not be assumed to be nonfunctional, but perhaps only less-fit in comparison to the protein functions encoded in nearby basins.

**Fig. 6:**
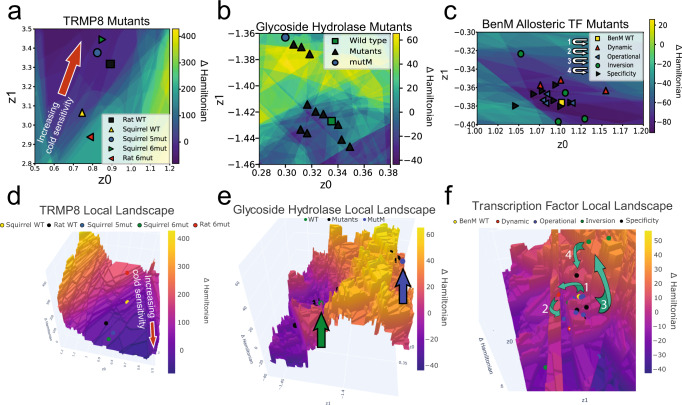
Application of local generative landscapes for protein engineering. **a**, **d** Transmembrane protein 8 (TRMP8) ion channel variants occupy different sequence space within a local basin. This difference is correlated with cold-sensitivity. Rat wild-type TRMP8 is cold sensitive while squirrel wild-type TRMP8 is not. Mutating 6 residues in the squirrel TRMP8 into those of the rat sequence induces cold-sensitivity and the reverse procedure diminishes cold-sensitivity in the rat 6 mutant sequence. Shown in 2-dimensions in (**a**) and 3-dimensions in (**d**). **b**, **e** Movement towards a basin can increase probability of generating a protein with specific properties. *β*-glucosidase D2-BGL mutants are engineered for higher saccharification efficiency. All mutants shown have similar or increased productivity to wild-type D2-BGL. Mutants near the wild-type show increased enzymatic efficiency, while mutM exhibits a 2.7 fold increase due to improvements in protein folding, sorting, and export efficiency in the endoplasmic reticulum. Shown in 2-dimensions in **b** and 3-dimensions in **e**. This illustrates how local fitness barriers divide sequence space with different functional properties. **c**, **f** Evolution-guided engineering of allosteric transcription factor, BenM, to recognize a new small molecule, adipic acid. Mutants showing increased gene expression over wild-type when treated with *cis,cis*-muconic acid are called dynamic range mutants (1). The dynamic mutant with asterisk is mutant MP02-G10, which exhibits the greatest increase in expression. Further mutation of dynamic mutants results in the operational range mutants (2) which exhibit similar levels of expression when treated with *cis,cis*-muconic acid and adipic acid as BenM. Further mutation of operational mutants results in inversion mutants (3) which now show higher expression when treated with adipic acid than *cis,cis*-muconic acid. Finally, mutation of inversion mutants results in specificity mutants (4) which, when treated with adipic acid, exhibit similar expression to BenM under *cis,cis*-muconic acid treatment. Shown in 2-dimensions in (**c**) and 3-dimensions in (**f**). This system further supports the notion that changes in selection pressure control escape from or pull towards basins. Rotational plots of 3D plots are available in Supplementary Fig. 10.

When considering local regions of the latent generative landscape, we can compare the landscape surrounding a sequence of interest using neighboring differences in Hamiltonian values, as calculated in Equation (10). This allows visualization of local, rugged sequence space. Transmembrane protein 8 (TRMP8) is an ion channel present in many organisms, but only important for cold thermosensation in a subset of organisms. Sequence diversity has been used to yield important information about the function and mechanism of these channels^69–71^. In the case of TRMP8, we are able to visualize how phenotypical information about cold-sensitivity is encoded in the LGL by using the wild-type, cold-sensitive rat sequence as the sequence of interest (Fig. 6a, d). When analyzing the non-cold-sensitive squirrel wild-type sequence, we see that it occupies a more positive (unfavorable) Hamiltonian space than the cold-sensitive wild-type rat sequence, hinting that the specific cold-sensing function of this channel is associated with a lower Hamiltonian value. Furthermore, we evaluate TRMP8 mutants generated by ref. ^69^. When the squirrel sequence is mutated in 6 key positions towards the amino acids present in the rat wild-type (H726Y, A762S, P819S, A927S, H946Y, and S947N), we see that this cold-sensitive variant moves towards the rat wild-type sequence and also to a more favorable relative Hamiltonian space. Similarly, when the rat wild-type sequence key residues are mutated to the squirrel amino acids, we see that the non-cold-sensitive rat mutant moves towards the squirrel wild-type sequence and into less favorable Hamiltonian space. For the squirrel 5-mutant, only 5 of the 6 key residues were changed into the rat amino acids (H726Y, A762S, A927S, H946Y, and S947N). This mutant had increased cold-sensitivity but was not as responsive as the squirrel 6-mutant. In Fig. 6d, the squirrel 6-mutant is slightly deeper into the basin of favorable Hamiltonian than the squirrel 5-mutant. Overall, we observe that mutants in more favorable fitness sequence space are correlated with the functional attribute of cold sensitivity. TRMP8 proteins that have the cold-sensitivity function move deeper into the local basin, while TRMP8 mutants lacking cold sensitivity move away from the local basin. This behavior shows how local basins encode preference for certain sequence attributes, including functions, and thus, sampling of basins or directing mutations to improve location relative to the local basin can assist in engineering of specific protein properties.

While movement towards a local basin in the latent generative landscape may help predict an attribute of mutants, we observe in the case of glycoside hydrolases that local fitness barriers may divide areas of sequence space with distinct properties. The glycoside hydrolase, D2-BGL, is of interest in material development due to its saccharification activity. In Kao, et al., D2-BGL mutants were generated to find variants with higher saccharification efficiency than wild-type D2-BGL^72^. Figure 6b, e shows wild-type D2-BGL and the increased productivity mutants generated. We observe that no mutants populate the peak of the local fitness barrier. Many mutants are clustered nearby wild-type D2-BGL in the local basin. Two of the key variants in the local basin were shown to have increased activity due to higher enzymatic efficiency. MutM, the highest productivity mutant, exhibited a 2.7 fold increase in expression, which was due to increased sorting, folding, and export from the endoplasmic recticulum while the other mutants nearby wild-type D2-BGL had increased expression due to increased enzymatic efficiency^72^. While all mutants exhibited increased productivity, we observe mutants crossing a local barrier of unfavorable fitness exhibit different properties than the proteins occupying the local basin. These local differences are often not discernible when only considering the global landscape (Supplementary Fig. 11b). This illustrates that local barriers can also divide sequence space into subsets with distinctive functional properties.

Accessing new or different properties is often the goal of protein engineering, but mutagenesis often leads to large libraries of nonfunctional mutants. To avoid destruction of important properties, rounds of positive and negative selection are interchanged to access new functional sequences^73–77^. One example of this is the toggled selection regime for allosteric transcription factor, BenM, to recognize a new ligand, adipic acid, from ref. ^78^. A mutant library was generated for BenM and nonfunctional mutants were removed, mutants with increased expression upon treatment with the cognate ligand, *cis,cis*-muconic acid, are designated as dynamic mutants. This mutational round is indicated by label (1) in Fig. 6f. When viewing dynamic mutants in the local LGL of allosteric transcription factors (PF03466), 2 of the 3 dynamic mutants occupy the local barriers around the BenM basin (Fig. 6c, f). The one dynamic mutant within the local basin, MP02-G10, exhibits a >15 fold improvement over wild-type BenM and outperforms the other dynamic mutants. This indicates that robust expression upon activation is associated with the local basin around BenM. This selection round has lower selection pressure, and we see most mutants occupying less favorable or neutral sequence space. The dynamic mutants are then used to create a secondary mutant library in which mutants are selected for similar expression to BenM after treatment with *cis,cis*-muconic acid and adipic acid, indicated by (2) in Fig. 6f. These mutants are designated operational range mutants and are shown to move back towards the local fitness basin. This supports the idea that increased selection pressure drives mutants toward landscape basins. Then, a tertiary library is generated from operational range mutants, indicated by (3) in Fig. 6f. Mutants showing higher expression with adipic acid than *cis,cis*-muconic acid are selected to create the inversion mutants. Inversion mutants undergo less selection pressure, exit the local basin, and occupy less favorable fitness space. Inversion mutants are then used to generate a quaternary mutant library where mutants are selected for comparable expression after treatment with adipic acid when compared to wild-type BenM treatment with *cis,cis*-muconic acid, indicated by (4) in Fig. 6f. Mutants selected from this final library are designated as specificity mutants. Specificity mutants once again move towards the local basin, indicating restoration of shared attributes that control enzymatic efficiency in wild-type BenM. These subsequent rounds of positive and negative selection are reflected in the local generative landscape, where lowered selection pressures allow movement of mutants toward less favorable or neutral sequence space, and high selection pressures enforce entrapment of mutants into the local basin. The occupation of higher diversity space under lowered selection pressure mimics behaviors observed in the Stiffler, et al. *β*-lactamase mutants (Fig. 5b(ii)). Toggling selection pressure for evolution-guided engineering has shown how sequences can move within and out of basins to gain specific attributes by accessing a different subset of sequence space.

### Functional annotation of sequences using LGLs

**Fig. 7:**
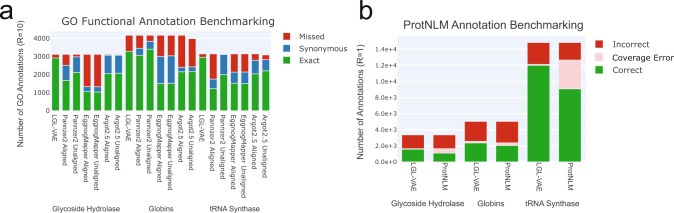
Comparison of LGL functional annotation performance and other sequence-based methods. **a** GO annotation predictions for glycoside hydrolase, globins, and tRNA synthetase were performed using the LGL, Pannzer2, Argot2.5, and eggNOG-mapper. Both aligned and unaligned sequences were used for other methods to ensure fair comparison. Annotations are considered exact if the test GO label matches the predicted GO label. Annotations are considered synonymous if the predicted GO label is either a child or parent of the test GO label. Annotations that are missed are test GO labels that were not predicted by the given method. **b** Annotation predictions for glycoside hydrolase, globins, and tRNA synthetase were performed by LGL and ProtNLM. Annotations are correct when they are either an exact match to the test protein or have the same meaning. Annotations with a coverage error are annotations where the correct label never appears in the predicted label set and the resulting prediction is not synonymous. Annotations are incorrect when the wrong annotation is selected.

For globins and *β*-lactamases, we proposed how the LGL could assist in the classification and analysis of uncharacterized proteins. To test the usefulness of the LGL for functional annotation of sequences, we compared LGL performance against current and also state-of-the-art methods (see *Methods* for details). Two separate protocols were used to compare the potential of LGL for annotation, the results are shown in Fig. 7. The first protocol focuses on functional annotation utilizing gene ontology (GO) terms, and the second compares annotations performed by the LGL and ProtNLM, which is a recently developed large language model^79^. GO annotations assigned using the LGL were comparable to those made by Pannzer2, Argot2.5, and eggNOG-mapper^80–82^, shown in Fig. 7a. LGL GO annotation of test sequences returned no synonymous predictions but performed marginally better than other methods at providing more specific GO labels, which were exact matches to the test sequence GO labels. To avoid effects of alignment of input sequences on performance, both unaligned and aligned input sequences were used for Argot2.5, Pannzer2, and eggNOG-mapper. Alignment of input sequences only had a noticeable impact on Pannzer2. The LGL had increased performance over eggNOG-mapper in all cases, but it is worth noting that eggNOG-mapper has a user friendly interface. Furthermore, the compared GO annotation methods do not require users to utilize their own compute resources. When comparing language model annotations between the LGL and ProtNLM, the LGL had comparable or marginally better performance for the protein families analyzed. We speculate in part because while ProtNLM had to learn and store labels using model parameters, the LGL was trained in an unsupervised way and retrieved prediction labels in a method more akin to a database of labels. One example of a common coverage error made by the language model is from the tRNA Synthetase family, where in our test set ProtNLM never produced the label “Aspartate–tRNA(Asp/Asn) ligase” and only predicted “Aspartate–tRNA(Asp) ligase”, despite these two labels conveying important functional specificity differences. These findings demonstrate that latent generative landscapes can also be reliably used for functional annotation of sequences.

### The latent landscape as an evolutionary map

**Fig. 8:**
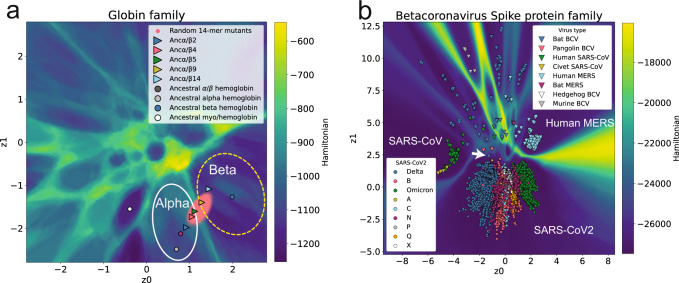
The VAE and landscape allow study and visualization of sequence evolution. **a** Ancestral sequences used for structural analysis and guided evolution labeled as Ancestral, and Ancestral *α*/*β* mutants chosen through structural analysis plotted with Anc prefix. Computationally generated 14-mer mutants plotted underneath. **b** The betacoronavirus family, colored separately by SARS-CoV2 lineage and viral type. Circles in this plot are all SARS-CoV2 lineage sequences, and the white arrow points at the RaTG13 Bat Coronavirus Spike sequence which has been suggested as the link between the animal and human SARS-CoV2.

In addition to functional classification and fitness, the LGL can be used as a tool to interpret and obtain insights on evolutionary trajectories. One example includes the engineering of ancestral hemoglobin sequences to gain functional heterotetramerization of alpha and beta hemoglobin sequence variants^83^. In brief, ancestral reconstruction was used to produce ancestral alpha/beta, alpha, and beta hemoglobin sequences, with the ancestral alpha/beta sequence having 41 amino acid substitutions compared to the beta sequence. The alpha/beta sequence was modified, swapping in residues from beta hemoglobin until it could form an *α*/*β* tetramer in solution with the ancestral alpha hemoglobin. The sequence gained tetramerization but was not soluble for native mass spectrometry at 9 mutations(*α*/*β*9), an additional set of mutations were required for native tetramerization (*α*/*β*14). Shown in Fig. 8a, when these sequences are encoded and shown in the landscape this transition can be explained through the coevolutionary information embedded in the VAE. The ancestral *α*/*β* hemoglobin encodes into the alpha hemoglobin basin (solid circle), and through successive mutation eventually crosses the landscape barrier and enters the beta hemoglobin basin (dashed circle), all of which is in line with the described experimental results. In this way, the landscape allows directed evolution experiments to be tested in silico in an unsupervised framework, where mutation from one sequence to another can be done at large scale and assessed within the landscape. In this example, there are 30 possible mutations between the aligned sequences of ancestral *α*/*β* and the *β* hemoglobin (Supplementary Table 1), and there are many combinations of 14-mer mutants that would allow the sequence to traverse the barrier and enter the beta hemoglobin basin (shown as a cloud of points in Fig. 8a). By counting the frequency of occurrence of positions used in synthetic 14-mers, the positions which push the encoded points towards the ancestral *β* hemoglobin often, but not always, agree with the original 14 chosen through structural analysis, Supplementary Figs. 12 and 13.

Qualitatively, the heights of these barriers can offer some indication of evolutionary distance between sequences. Shown in Fig. 8b, when plotting an alignment of spike proteins from the coronavirus family of viruses, the heights of the barriers coincide with established phylogenetic relationships between family members^84^. With the LGL it is clear that straight line movement from the SARS-CoV2 cluster to the Human MERS cluster entails breaking many more of the sequence couplings than a similar distance from the SARS-CoV2 cluster to the SARS-CoV cluster, highlighting the evolutionary distances between these three betacoronaviruses. The SARS-CoV2 cluster contains all unique sequences from the NCBI SARS-CoV2 repository^85^ (accessed on March 31st, 2022), and they are distributed radially around the earliest SARS-CoV2 sequences deposited, which can be seen more clearly in Supplementary Video 1. The map also shows how there is no clear barrier between the Bat Coronavirus sequence (white arrow) and the large number of variants sequenced during the pandemic, consistent with the notion that the Bat might be the link between human and animal SARS-Cov2.

## Discussion

While previous work has found that the intrinsic dimension of protein sequence datasets is close to 10^86^, in this work, we show how 3 dimensions (2 latent variables and the DCA Hamiltonian) in the latent generative landscape provides greater interpretability of the VAE manifold and useful information on protein sequence properties which would be more difficult to achieve with higher dimensions. As shown in Figs. 3a, 5a and 8, phylogenetic and functional differences can be easily identified using the LGL. The simplest information gain is that high Hamiltonian barriers can be considered as a demarcation between groups of proteins. In previous methods, classifying sequences using a VAE required clustering analysis, but by using Hamiltonian barriers instead, we could utilize biologically relevant information to inform classification of sequences. This could reduce errors when a sequence seems to be equidistant to two clusters of known function and/or phylogeny, but in reality there exists a coevolutionary (Hamiltonian) barrier between them as shown in Fig. 8a. In Fig. 3, for example, unlabeled sequences encoding near the barrier between myoglobin and hemoglobin subunit *α* would be difficult to classify with distance based clustering analysis alone, yet with the LGL barrier this becomes significantly easier and more reliable. Classification based on landscape barriers seems a promising method, Fig. 3b, and may provide advantages over other methods when classifying sequences. Furthermore, the LGL allows further subclassification of sequence space that cannot be achieved through clustering alone. By identifying subsets of sequence space divided by weak Hamiltonian barriers, such as the separation between TEM and SHV *β*-lactamases in Fig. 5b(i), we are able to distinguish between functionally different groups. In the case of TEM and SHV, identifying whether a class-A *β*-lactamase belongs to either group is usually done experimentally as even their structures are incredibly similar^64^. Thus, the LGL provides a powerful tool for classifying new variants in silico.

It is important to also recognize that the latent generative landscape quantifies the attributes of all sequence space, not only the proteins within the training set. Thus, the underlying landscape can be rationally sampled to select for certain traits when generating de novo proteins. Sampling from basins is recommended because, as shown by encoding of higher order marginals in Fig. 4, these regions tend to have higher fidelity to input sequence statistics. This means that the likelihood of generating a functional sequence may be higher in basins than in barriers^15^. While it is true that the regions of the VAE landscape which have a higher Hamiltonian could still be expressed and produce valid proteins (Supplementary Figs. 14 and 15), there is good evidence that the Hamiltonian energy has predictive power for the functional viability of an expressed protein^23,26^. Moreover, we observe that regions near the origin have high entropy and low adherence to family statistics, Fig. 4 and Supplementary Fig. 8. While it is possible that this sequence space contains useful information, such as the sequence of family origin^32^, reconstructing any sequence of relevance to the family might be more challenging and computationally expensive due to high Shannon entropy of decoded sequences. Thus, sampling the origin could produce an extraordinary number of unique sequences, in which many do not adhere to family statistics as indicated by Fig. 4. The entropy affects the Hamiltonian scores of sequences generated through evaluating decoded distributions, as shown in Supplementary Figs. 16 and 17, highlighting that this variability is inversely related to the distance from the central region of the landscape. When observing Hamiltonian barriers outside of the origin, we observe varying heights. Thus, we propose that some barriers have a higher cost to cross than other barriers. High cost of traversal between more distantly related sequences was also demonstrated in Fig. 8b.

With the latent generative landscape defining subsets of sequence space and cost of fitness to access new functions, we can also consider smaller, local differences encoded in the landscape. In the case of TRMP8, cold-sensitive sequences are encoded deeper into basins. This includes mutant sequences, which were not present in the training set, Fig. 6a, d. This example demonstrates how proteins can be mutated toward new functions using the LGL. It also suggests that this framework can be used to rationally sample sequence space in attempt to preserve specific attributes, such as cold-sensitivity. When considering local differences, we also see how local barriers subdivide sequence space in the case of glycoside hydrolase, Fig. 6b, e. Functionally improved mutants, which were not in the training set, cluster near wild-type D2-BGL in a basin when their increase in function is associated with increased enzymatic efficiency. MutM shows increased performance due to more efficient folding and export from the endoplasmic reticulum to the Golgi body, as determined by changes in expression of unfolded protein response (UPR) genes^72^. This supports the idea that proteins with similar functions are encapsulated in local basins and that even relatively small barriers can indicate sequence space with differing attributes. Generating sequences from within the basin may give specific enzymatic properties, but mutating sequences to different local basins may allow new attributes to be gained. Through these examples, we show how highly-specialized mutants could be more reliably generated, screened, and designed using the LGL.

Interestingly, the LGL could also be useful in modeling evolutionary trajectories. In Fig. 8a, the process of hand selecting the mutants used in ref. ^83^ required expert knowledge and a wealth of prior information on the structure and function of hemoglobin, whereas a similar result can be found by a less informed but more exhaustive, in silico assessment of mutations to create variants with potentially the desired function. This could lead to a method for unsupervised assessment of directed evolution in proteins with less supporting information. Additionally, in Figs. 5b(ii, iii) and 6c, f, we see how lowered selection pressure allows directed-evolution mutants to occupy less favorable sequence space and cross Hamiltonian barriers while directed-evolution mutants under higher selection pressure move further into basins. In these cases, we observe how lowered selection pressure allows mutants to occupy sequence space that is otherwise not accessible. Multiple rounds of varying pressure also seem to influence ability of sequences to occupy less favorable spaces, but this maybe be attributed to robustness encoded in the protein family. We can assume that protein families with higher sequence diversity also allow for less favorable sequences to more easily persist, as it has proven advantageous. The circular movement of mutants during a toggled selection regime (see Fig. 6f) is reminiscent of the re-encoding of generated sequences as shown in Fig. 1b and Supplementary Fig. 18. These flows show a striking correspondence with the landscape, and intriguingly, the direction of the arrows implies that there are many regions within these maps where sequences which are generated from the decoder do not change their position upon re-encoding. Perhaps these flows capture some evolutionary behavior that is controlled by selection pressure. VAEs have been connected to concepts like attractors before^87^, though in our example, the effect is not due to intentional model over-fitting, and so in the context of phylogenetics these basins of attraction may have useful similarities with the ancestral reconstruction method of sequences through phylogenetic relationships. Further investigation of flows can help identify if this phenomenon is only an attribute of the model or indicative of some real, evolutionary behavior.

Altogether, we have presented several examples in which the LGL could be used to inform selection of coordinates for generation of de novo proteins by avoiding sequence space with poor Hamiltonian values. This may improve generation of functional proteins for VAEs and VAE-related architectures, which have faced challenges in the past^34,39,40^. Sampling from basins may also be useful in selecting for variants with specific functions. We have shown the present methodology could be used to generate and screen mutants for properties that are defined by landscape basins. This allows for in silico testing where libraries are generated computationally and then specific variants are selected based upon their LGL coordinates. The landscape itself may also be useful for understanding how and why mutants are able to access new sequence space and what sequence features allow for neofunctionalization. Thus, our next steps include the further use of machine learning methods on coevolutionary information to further improve in silico protein engineering and generation, as well as explore and define relevant evolutionary behaviors.

## Methods

### Pfam sequence collection

HMMSearch^49^ against the Uniprot database was used to obtain MSAs for the protein families Globin (PF00042), FA_desaturase (PF00487), Beta-lactamase2 (PF13354), Acetyltransf_1 (PF00583), and LysR_substrate (PF03466) using the PFAM HMM seed^48^. Seed sequences were used for TRMP8 and glycoside hydrolase due to poor coverage of PFAM domain with experimental mutational sites and are available in the Datadryad repository. Number of training sequences for each family is shown in Supplementary Table 2.

### Betacoronavirus spike sequence collection

Seed sequences were full length spike proteins which were aligned so that all human SARS-CoV2 spike protein positions were included (L = 1274). This seed MSA was used to perform an HMMSearch on the Uniprot database. SARS-CoV2 sequences were pulled from NCBI on March 31st, 2022, and were aligned to the seed HMM with hmmalign. All duplicate sequences were removed before training. This final MSA was used to train a VAE model which had 2548 hidden units. All SARS-CoV2 label information was parsed programmatically from the *json* file which accompanied the NCBI data deposition, and all other sequences were labeled with information from their respective Uniprot headers.

### Data pre-processing

The collected MSAs were filtered to remove sequences with 20% or greater contiguous gaps. Sequences of length *N* were one hot encoded with gaps encoded as an additional character. One hot encoding is the procedure in which data is converted into a binary matrix. For the model, each aligned sequence, *x*, is a 23 by *L* matrix. Rows encode all possible amino acid characters, including a gap character, and *L* is the length of *x*. When analyzing mutant libraries, mutants with a nonsense mutation of over 20% the original protein length were discarded.

### VAE model architecture

The design goal of the variational autoencoder is to generate data samples *x* ∈ *X* using a latent variable model defined on some parameters *θ* with a prior *p*_*θ*_(*z*) on latent variables *z*, such that the marginal likelihood is equal to

1 $${p}_{\theta }(x)=\int{p}_{\theta }(x|z)d{p}_{\theta }(z)$$

The parameters *θ* and the latent variables **z** are not known, and this form does not give a tractable algorithmic solution for finding them. The solution proposed in ref. ^36^ is to approximate the posterior distribution *p*_*θ*_(**z**∣**x**) with another model defined on parameters *ϕ*,

2 $${q}_{\phi }({{{{{{{\bf{z}}}}}}}}|{{{{{{{\bf{x}}}}}}}})$$

This model defined on *ϕ* is termed the encoder, and the model defined on *θ* is termed the decoder. The marginal likelihood of generating a sample *x* through the decoder can now be written as

3 $$\log {p}_{\theta }({{{{{{{\bf{x}}}}}}}})={D}_{KL}({q}_{\phi }({{{{{{{\bf{z}}}}}}}}|{{{{{{{\bf{x}}}}}}}})||{p}_{\theta }({{{{{{{\bf{z}}}}}}}}|{{{{{{{\bf{x}}}}}}}}))+{{{{{{{\mathcal{L}}}}}}}}(\theta,\phi,{{{{{{{\bf{x}}}}}}}})$$

where *D*_*KL*_ is the Kullback-Leibler divergence, measuring the fit between the decoder’s posterior distribution on *z* and the encoder’s posterior, and the last term ${{{{{{{\mathcal{L}}}}}}}}$ is the lower bound of the model’s fit to the marginal distribution over *z*. Equation (3) can be rewritten into the evidence lower bound function (ELBO)

4 $${{{{{{{\rm{ELBO}}}}}}}}=-{E}_{{{{{{{{\bf{z}}}}}}}} \sim {q}_{\phi }({{{{{{{\bf{z}}}}}}}}|{{{{{{{\bf{x}}}}}}}})}\log ({p}_{\theta }({{{{{{{\bf{x}}}}}}}}|{{{{{{{\bf{z}}}}}}}}))+{D}_{KL}({q}_{\phi }({{{{{{{\bf{z}}}}}}}}|{{{{{{{\bf{x}}}}}}}})||{p}_{\theta }({{{{{{{\bf{z}}}}}}}}))$$

which is the objective function minimized during training. On the right-hand side, the first term is the reconstruction error which measures how well the encoded data matches the generated data (minimum of zero), and the second term is the measure of fit of the latent distribution which is being encoded and an assumed prior distribution (minimum of zero). We follow^36^ and encode the posterior *q*_*ϕ*_(**z**∣**x**) using a reparameterization procedure to ensure our model to be differentiable. The encoder model encodes sequences as Gaussian parameters *μ* and *σ*^2^, and these parameters are combined through an element-wise matrix product with an auxiliary noise variable *ϵ*, such that

5 $$z=\mu+\sigma \odot \epsilon$$

This reparameterized *z* is the code that the decoder uses to generate sequences, and this lets us define *p*_*θ*_(**z**) as a Gaussian distribution to give the gradient of Equation (4) an analytical solution. For our specific implementation, the data is represented as a one hot encoded vector, where for a protein of length *L* we create an array of shape 23 × *L*, with each row containing a 1 in a position linked to an amino acid identity with the remaining row positions containing a 0. A total of 23 rows were used to encode the 20 canonical amino acids, a gap character, and additional less common amino acids. The latent variables **z** are decoded into a *Softmax* probability distribution with the same array dimensions as the input, where the output layer $\psi :{{\mathbb{R}}}^{23\times L}$ with each column corresponding to 23 sequence symbols (*a* ∈ *A*) is measured as

6 $$p{(a|{{{{{{{\bf{z}}}}}}}})}_{i}=\frac{\exp ({\psi }_{{a}_{i}}({{{{{{{\bf{z}}}}}}}}))}{{\sum }_{k\in A}\exp ({\psi }_{{k}_{i}}({{{{{{{\bf{z}}}}}}}}))}$$

which gives *L* rows with probability values summing to one in each row. The reconstruction error term in Equation (4) will evaluate as zero if the input and output matrices are identical (i.e., the only sequence possible at some point *z* is the input sequence).

### Hyperparameters and training

For all of our models, unless stated otherwise, we used 3 × *L* hidden units for both the encoder and decoder, the ReLU activation function for these hidden units, a latent dimension of 2, the Adam optimizer with a learning rate of 1*e* − 4, and a *l*2-regularization penalty on the hidden units of 1*e* − 4. Training was stopped when loss did not improve within 10 epochs. With only 2 latent encoding dimensions we saw no improvement in test set validation when using more than 3 × *L* hidden units. Models were built using *Tensorflow*^88^ and trained either on workstations or on NVIDIA A100 GPUs.

### Landscape generation

For a given trained VAE model, the input training sequences used to create the VAE are used to generate a Direct Coupling Analysis (DCA) model^3^, defined as

7 $$P(S)=\frac{1}{Z}\exp \left\{\mathop{\sum}\limits_{i < j}{e}_{ij}({A}_{i},{A}_{j})+\mathop{\sum}\limits_{i}{h}_{i}({A}_{i})\right\}$$

which defines the probability of a sequence *S* of length *L*, defined by the statistics of occuring amino acids at single positions *A*_*i*_ and pairs of positions (*A*_*i*_, *A*_*j*_). The *e*_*ij*_ term are parameters related to the pairwise couplings between MSA positions and the *h*_*i*_ is a local field related to the frequency of amino acids at that position. The parameters of this model can be inferred in different ways^4,89,90^, here we used the inverse of the cross-correlated matrix as described in ref. ^3^. In a grid-like fashion, the VAE’s decoder is fed with uniformly spaced coordinates (*z*_0_, *z*_1_) to generate a decoded *Softmax* distribution as described in Equation (6). The maximum probability sequence from this output distribution is generated as

8 $${S}^{*}({{{{{{{\bf{z}}}}}}}})={a}_{i}\ldots L,\quad {{{{{{{\rm{where}}}}}}}}\,\,{a}_{i}=\arg \mathop{\max }\limits_{a\in A}p{(a|{{{{{{{\bf{z}}}}}}}})}_{i}$$

Each sequence is given a Hamiltonian score using the parameters obtained from the Boltzmann-like DCA distribution, defined as follows:

9 $$H({S}^{*})=-\mathop{\sum}\limits_{1\le i < j\le L}{e}_{ij}({a}_{i},{a}_{j})-\mathop{\sum }\limits_{i=1}^{L}{h}_{i}({a}_{i})$$

### Delta Hamiltonian score

We can also quantify and create landscapes with the difference between Hamiltonian scores using a reference sequence. This Δ Hamiltonian is defined as

10 $$\Delta {H}_{{S}_{n}}={H}_{{S}_{n}}-{H}_{{S}_{ref}}$$

where *S*_*n*_ can be either a generated sequence or a mutated sequence while *S*_*ref*_ can be a wild-type or extant sequence or a reconstructed wild type in the case of local landscapes, where all sequences (including wild-type) used to construct the LGL are in their decoded representation S^*^. The landscape map is then created with the collection of latent coordinates *z*_0_, *z*_1_ and the value of the Hamiltonian for each of the sequences generated using Equation (8).

### Tree topology comparison

For Fig. 3b(i, ii), a Gaussian mixture model was used with 70 clusters to separate the encoded training data arbitrarily into groups. We chose 70 clusters in order to fit Gaussian distributions that did not overlap with barriers and did not heavily fragment the clustering in the landscape, and to this end, we additionally removed 6 of the Gaussians, which spanned across barriers to leave us with 64 distributions. The mean and variances of the distributions were used to generate points, and those points were first filtered to ensure they did not lie on barriers (a Hamiltonian cutoff at −850 was used for the Globin family), and were then used as input into the decoder to produce a probability distribution which was evaluated to generate a sequence. For each cluster, 10 sequences were generated and 10 natural sequences were randomly chosen and each was assigned a group number label and a number 0–9, with each MSA totaling 640 sequences. This allows generated sequences to be matched to real sequences where topological similarity implies phylogenetic correspondence of VAE sequences and extant sequences. For Fig. 3b(iii), no clustering was used, and instead the Globin (PF00042) and FA_Desaturase (PF00487) Pfam MSA were sampled. The full MSAs were used, and when sampled, their identity was set to their taxonomic ID number given by Uniprot so that tree similarity would be defined by taxonomic organization. A total of 100 of these MSA pairs were generated, each with 640 sequences to ensure comparable scores. All MSAs created were computed into phylogenetic trees using the FastTree software^91^ set to default settings. The metric used to compare trees was the Clustering Information Distance (CID) from the TreeDist R package^58^, which measures the similarity in leaf arrangement between two trees. This method finds optimal matching between split points on two trees being compared (each tree is split into two subtrees), then uses a mutual information score to measure the information difference between the two trees based on the leaf labels in their respective subtrees given by the split. This is done for all optimal tree splits given two trees and summarized as a distance score. If the trees are identical their clustering distance is zero, and for completely random trees their score increases in proportion to their size. For Fig. 3b(iv), the natural Globin trees generated for subfigure Fig. 3b(i) had their leaves shuffled randomly and were compared to the ordered trees. This shows the expected null score from this metric at these tree sizes.

### VAE sampling for r20 scoring

For Fig. 4, the Globin (PF00042) Pfam family was used to train a VAE model, and a 500 × 500 element (pixels) landscape was generated. Coordinates were generated with a ${{{{{{{\mathcal{N}}}}}}}}(0,2{{{{{{{\bf{I}}}}}}}})$ distribution. These coordinates were fed into the decoder, producing probability distributions whose values were used to generate two sequences, one sequence through evaluating the probability distribution and a second through Equation (8). For each coordinate, if the sequence created through Equation (8) had a Hamiltonian score below the specified cutoff, the probability evaluated sequence was added to the dataset, and this process was repeated until 10,000 sequences were generated. These filtered MSAs were compared to the training data using the *r20* correlation score used in ref. ^55^. For each *K* ∈ {2, 3, 4, 5, 6, 7} the K-site marginal statistics were compared between the generated sequences and the input training sequences. For each K-mer, 3000 unique sets of *K* columns from the training MSA were chosen and all unique sequence motifs in these positions were counted and normalized with the top 20 most common motifs being chosen for comparison. The same columns were chosen in the generated MSAs, and the selected 20 motifs were counted and normalized. These normalized frequencies were compared through a Pearson correlation score to measure the agreement between the *K*-site statistics in the real sequences and the synthetic sequences, with a score of 1 meaning perfect agreement. These 20 correlations were averaged to produce the final value for each K-site point in each generated MSA.

For the sequence statistics analysis shown in Supplementary Fig. 3, we follow, with some differences, the method described in ref. ^55^ for producing the four Pfam derived datasets for PF00005, PF00069, PF00072, and PF00076. Seed sequences from Pfam were downloaded and queried against the Uniprot database (2021_02) using HMMSearch. From these aligned sets we filter out sequences with greater than 20% contiguous gaps, then randomly sample from this dataset, each time removing sequences from the sample pool with sequence identity greater than 55% to the newly sampled sequence. When 20,000 sequences are selected, we split this set randomly into 10,000 train and 10,000 test sequences. We train VAE models with two and seven latent dimensions on the full training sets as described in the Hyperparameters and Training section, with no holdout validation sets. Sequence generation and *r20* evaluation was performed as described above, with no cutoffs used. These sequences were compared to the testing set to produce the reported data.

### Ancestral Globin plot

The VAE was trained using the Globin Pfam MSA (PF00042). The deposited Ancestral Globins and mutants from ref. ^83^ were aligned to the Globin Pfam MSA using the HMM from Pfam before being encoded. There were 39 sequence differences between the Ancestral *α*/*β* Hemoglobin and the Ancestral *β* Hemoglobin, and the *α*/*β* Hemoglobin was the template for mutation. For each mutant, 14 of the 39 positions were chosen and those positions were mutated to match the *β* Hemoglobin sequence. We generated 10,000 of these mutants and plotted them in Fig. 8a.

### Streamplot

The streamplot was created using *matplotlib*^92^. For each 2-D coordinate input in a grid-like fashion in order to generate the latent generative landscape, a maximum probability sequence was generated through Equation (8), and this sequence was subsequently fed into the encoder portion of the VAE to generate a new latent coordinate (*z*_1_, *z*_2_). The initial pixel coordinates and the resulting encoded coordinates becomes the set of vectors used to create the streamplot. Both streamplots were made with the Globin VAE model described in earlier Methods.

### Latent space entropy calculation

Entropy landscape is calculated in a grid-like fashion using decoder distribution (X) at each position in the landscape, where average entropy per amino acid at any given coordinate is termed $\hat{H},L$ is the length of the protein, *i* is the residue position, and *q* is each amino acid character possibility.

11 $$\hat{H}(X)=-\frac{1}{L}\mathop{\sum }\limits_{i=1}^{L}\mathop{\sum }\limits_{q=1}^{23}P\left({x}_{i}^{q}\right)\log \left(P\left({x}_{i}^{q}\right)\right)$$

### Functional annotation comparisons

For the ProtNLM comparison, we query Uniprot release 2022_04 using seed sequences for Globin (PF00042), tRNA Synthetase (PF00152), and Glycohydrolase (single seed sequence), then split the data into train and test sets using sequences which were added to Uniprot before the 2021_02 release as the training set to train two dimensional VAE models for each family, mimicking the temporal split used to train ProtNLM^79^. The resulting splits are: 10,218 Globin training sequences and 5037 Globin test sequences, 27,164 Glycohydrolase training sequences and 3357 Glycohydrolase test sequences, and 98214 tRNA Synthetase training sequences and 14845 tRNA Synthetase test sequences. The prediction target labels for both methods are the Uniprot Protein names associated with each test sequence. ProtNLM was run on an NVIDIA 3090 using the freely available pretrained model and code in order to predict labels for the test sequences, taking the top scoring prediction. For the VAE, test sequences were encoded into the LGL and the nearest training sequence to the encoded test sequence had its sequence label pulled as the prediction label. For both VAE and ProtNLM methods we made our best attempt to match ambiguous labels, for example, if the Uniprot derived truth label is “Hemoglobin Subunit Beta” and ProtNLM predicts “HBBProtein” this is considered “Correct”. These matches are combined with exact matches to produce the “Correct” score. Predictions which fail to match are assessed to see if the truth label being predicted existed within the set of all prediction labels produced for the test set. If the ground truth label did not exist in the set of all generated prediction labels, then this is considered a “Coverage Error”; all other errors are listed as “Incorrect”. For the 351 Globin ground truth labels, ProtNLM learned 82 and the LGL received 260 through the training data. For the 169 Glycohydrolase ground truth labels, ProtNLM learned 12 and the LGL received 166 through the training data. For the 159 tRNA Synthetase ground truth labels, ProtNLM learned 34 and the LGL received 152 through the training data.

For Gene Ontology annotation benchmarking, the LGL was tested against three sequence-based methods: Pannzer2, Argot2.5, and eggNOG-mapper^80–82^. Pannzer2 is a weighted k-nearest neighbors (KNN) based on sequence similarity and enrichment^80^. Argot2.5 utilizes a weighting algorithm for homologous sequence GO annotations^81^. EggNOG-mapper utilizes precomputed phylogenies and orthologs to assign GO annotations^82^. GO annotations were collected using QuickGO API and only GO annotations with experimental evidence were used^93,94^. There were 314 annotated Globins, 577 annotated Glycohydrolases, and 451 annotated tRNA Synthetases. For each annotated protein, multiple annotations frequently exist, usually describing separate features of each protein. There were 2216 total experimental globin GO annotations, 1731 total experimental glycoside hydrolase GO annotations, and 1724 total experimental tRNA synthetase GO annotations. Proteins with GO annotations were split into 90% training and 10% test randomly 10 times. Training GO annotations were used to assign test GO annotations using nearest neighbor in VAE latent space. Testing set sequences were used as input to the LGL, Pannzer2, Argot2.5, and eggNOG-mapper. Since all methodologies require a homology search, both unaligned and aligned inputs were input to Pannzer2, Argot2.5, and eggNOG-mapper to ensure fair comparison to the LGL. Since GO annotations exist in trees where leaf nodes are more descriptive, parent and child nodes are considered synonymous.

### Reporting summary

Further information on research design is available in the Nature Portfolio Reporting Summary linked to this article.

## Supplementary information

Supplementary Information

Description of Additional Supplementary Files

Supplementary Movie 1

Reporting Summary

## Data Availability

The sequence, model, and validation data generated in this study have been deposited in the DataDryad database under accession code [10.5061/dryad.51c59zwbn]^95^. The processed HMM seed data are available at PFAM on InterPro [https://www.ebi.ac.uk/interpro/]^48^. The unprocessed sequence data are available at Swiss-Prot and TREMBL on Uniprot [https://www.uniprot.org/]^96^. The plotting data generated in this study are provided in the Source Data file are available in the DataDryad database.

## Code availability

An Interactive tool to generate and visualize LGLs (Supplementary Fig. 19) accompanied by code, scripts, and the Dockerfile used in this work is available on Github (https://github.com/morcoslab/LGL-VAE/)^97^.

## References

[1] Onuchic JN, Wolynes PG (2004). Theory of protein folding. Curr. Opin. Struct. Biol..

[2] Orengo CA, Thornton JM (2005). Protein families and their evolution-a structural perspective. Annu. Rev. Biochem..

[3] Morcos F (2011). Direct-coupling analysis of residue coevolution captures native contacts across many protein families. Proc. Natl Acad. Sci. USA.

[4] Ekeberg M, Lövkvist C, Lan Y, Weigt M, Aurell E (2013). Improved contact prediction in proteins: Using pseudolikelihoods to infer potts models. Phys. Rev. E.

[5] Ovchinnikov S, Kamisetty H, Baker D (2014). Robust and accurate prediction of residue-residue interactions across protein interfaces using evolutionary information. eLife.

[6] Hopf TA (2019). The evcouplings python framework for coevolutionary sequence analysis. Bioinformatics.

[7] Sułkowska JI, Rawdon EJ, Millett KC, Onuchic JN, Stasiak A (2012). Conservation of complex knotting and slipknotting patterns in proteins. Proc. Natl Acad. Sci..

[8] Marks DS (2011). Protein 3d structure computed from evolutionary sequence variation. PLoS One.

[9] Baek M (2021). Accurate prediction of protein structures and interactions using a three-track neural network. Science.

[10] Jones DT, Buchan DWA, Cozzetto D, Pontil M (2011). PSICOV: precise structural contact prediction using sparse inverse covariance estimation on large multiple sequence alignments. Bioinformatics.

[11] dos Santos RN, Morcos F, Jana B, Andricopulo AD, Onuchic JN (2015). Dimeric interactions and complex formation using direct coevolutionary couplings. Sci. Rep..

[12] Karmi O (2017). Interactions between mitoneet and naf-1 in cells. PLoS One.

[13] Hopf TA (2014). Sequence co-evolution gives 3d contacts and structures of protein complexes. elife.

[14] Quignot C (2021). Interevdock3: a combined template-based and free docking server with increased performance through explicit modeling of complex homologs and integration of covariation-based contact maps. Nucleic Acids Res..

[15] Cheng RR, Morcos F, Levine H, Onuchic JN (2014). Toward rationally redesigning bacterial two-component signaling systems using coevolutionary information. Proc. Natl Acad. Sci. USA.

[16] Sinner C, Ziegler C, Jung YH, Jiang X, Morcos F (2021). Elihksir web server: Evolutionary links inferred for histidine kinase sensors interacting with response regulators. Entropy (Basel, Switz.).

[17] Zhou Q (2018). Global pairwise rna interaction landscapes reveal core features of protein recognition. Nat. Commun..

[18] Bitbol A-F, Dwyer RS, Colwell LJ, Wingreen NS (2016). Inferring interaction partners from protein sequences. Proc. Natl Acad. Sci..

[19] Gueudré T, Baldassi C, Zamparo M, Weigt M, Pagnani A (2016). Simultaneous identification of specifically interacting paralogs and interprotein contacts by direct coupling analysis. Proc. Natl Acad. Sci..

[20] Dimas RP, Jiang X-L, de la Paz JA, Morcos F, Chan CTY (2019). Engineering repressors with coevolutionary cues facilitates toggle switches with a master reset. Nucleic Acids Res..

[21] Frazer J (2021). Disease variant prediction with deep generative models of evolutionary data. Nature.

[22] Rodriguez-Rivas J, Croce G, Muscat M, Weigt M (2022). Epistatic models predict mutable sites in sars-cov-2 proteins and epitopes. Proc. Natl Acad. Sci..

[23] Jiang XL, Dimas RP, Chan CTY, Morcos F (2021). Coevolutionary methods enable robust design of modular repressors by reestablishing intra-protein interactions. Nat. Commun..

[24] Tutol JN (2021). A single point mutation converts a proton-pumping rhodopsin into a red-shifted, turn-on fluorescent sensor for chloride. Chem. Sci..

[25] Chi H (2022). Coupling a live cell directed evolution assay with coevolutionary landscapes to engineer an improved fluorescent rhodopsin chloride sensor. ACS Synth. Biol..

[26] Russ WP (2020). An evolution-based model for designing chorismate mutase enzymes. Science.

[27] de la Paz JA, Nartey CM, Yuvaraj M, Morcos F (2020). Epistatic contributions promote the unification of incompatible models of neutral molecular evolution. Proc. Natl Acad. Sci. USA.

[28] Jumper J (2021). Highly accurate protein structure prediction with alphafold. Nature.

[29] AlQuraishi M (2019). End-to-end differentiable learning of protein structure. Cell Syst..

[30] Du X (2017). Deepppi: Boosting prediction of protein-protein interactions with deep neural networks. J. Chem. Inf. Model..

[31] Tubiana J, Cocco S, Monasson R (2019). Learning protein constitutive motifs from sequence data. eLife.

[32] Ding, X., Zou, Z., & Brooks, C. L. Deciphering protein evolution and fitness landscapes with latent space models. *Nat. Commun.* **10**(1), 5644 (2019). 10.1038/s41467-019-13633-0 PMC6904478 31822668

[33] Riesselman AJ, Ingraham JB, Marks DS (2018). Deep generative models of genetic variation capture the effects of mutations. Nat. Methods.

[34] Greener JG, Moffat L, Jones DT (2018). Design of metalloproteins and novel protein folds using variational autoencoders. Sci. Rep..

[35] Sgarbossa D, Lupo U, Bitbol A-F (2023). Generative power of a protein language model trained on multiple sequence alignments. eLife.

[36] Kingma, D. P. & Welling, M. Auto-encoding variational bayes. In *Proc. 2nd International Conference on Learning Representations, ICLR* 12 (2013).

[37] Kingma, D. P. & Welling, M. An introduction to variational autoencoders. arXiv.org (2019).

[38] Park, S. & Kim, H. Facevae: Generation of a 3d geometric object using variational autoencoders. *Electronics* **10**, 2792 (2021).

[39] Dean SN, Walper SA (2020). Variational autoencoder for generation of antimicrobial peptides. ACS Omega.

[40] Hawkins-Hooker A (2021). Generating functional protein variants with variational autoencoders. PLOS Comput. Biol..

[41] Dai B, Wang Y, Aston J, Hua G, Wipf D (2018). Connections with robust pca and the role of emergent sparsity in variational autoencoder models. J. Mach. Learn. Res..

[42] Tian H (2021). Explore protein conformational space with variational autoencoder. Front. Mol. Biosci..

[43] Grønbech CH (2020). scvae: variational auto-encoders for single-cell gene expression data. Bioinformatics.

[44] Nissen JN (2021). Improved metagenome binning and assembly using deep variational autoencoders. Nat. Biotechnol..

[45] Hong Y, Lee J, Ko J (2022). A-prot: protein structure modeling using msa transformer. BMC Bioinforma..

[46] Brandes N, Ofer D, Peleg Y, Rappoport N, Linial M (2022). Proteinbert: a universal deep-learning model of protein sequence and function. Bioinformatics.

[47] Repecka D (2021). Expanding functional protein sequence spaces using generative adversarial networks. Nat. Mach. Intell. 2021 3:4.

[48] Finn RD (2013). Pfam: the protein families database. Nucleic Acids Res..

[49] Eddy SR (2011). Accelerated profile hmm searches. PLoS Comput. Biol..

[50] Levy RM, Haldane A, Flynn WF (2017). Potts hamiltonian models of protein co-variation, free energy landscapes, and evolutionary fitness. Curr. Opin. Struct. Biol..

[51] Jacquin H, Gilson A, Shakhnovich E, Cocco S, Monasson R (2016). Benchmarking inverse statistical approaches for protein structure and design with exactly solvable models. PLOS Comput. Biol..

[52] Cheng RR (2016). Connecting the sequence-space of bacterial signaling proteins to phenotypes using coevolutionary landscapes. Mol. Biol. Evolut..

[53] Figliuzzi M, Jacquier H, Schug A, Tenaillon O, Weigt M (2015). Coevolutionary landscape inference and the context-dependence of mutations in Beta-Lactamase TEM-1. Mol. Biol. Evolut..

[54] Bisardi M, Rodriguez-Rivas J, Zamponi F, Weigt M (2022). Modeling sequence-space exploration and emergence of epistatic signals in protein evolution. Mol. Biol. Evolut..

[55] McGee F (2021). The generative capacity of probabilistic protein sequence models. Nat. Commun..

[56] Wright S (1932). The roles of mutation, inbreeding, crossbreeding, and selection in evolution. Proc. Sixth Int. Congr. Genet.,.

[57] Mettananda S, Gibbons RJ, Higgs DR (2016). Understanding a-globin gene regulation and implications for the treatment of b-thalassemia. Ann. N. Y. Acad. Sci..

[58] Smith MR (2020). Information theoretic generalized Robinson-Foulds metrics for comparing phylogenetic trees. Bioinformatics.

[59] Detlefsen NS, Hauberg S, Boomsma W (2022). Learning meaningful representations of protein sequences. Nat. Commun..

[60] Tooke CL (2019). *β*-lactamases and *β*-lactamase inhibitors in the 21st century. J. Mol. Biol..

[61] Bush K (2018). Past and present perspectives on *β*-lactamases. Antimicrob. Agents Chemother..

[62] Palzkill T (2018). Structural and mechanistic basis for extended-spectrum drug-resistance mutations in altering the specificity of tem, ctx-m, and kpc *β*-lactamases. Front. Mol. Biosci..

[63] Liakopoulos A, Mevius D, Ceccarelli D (2016). A review of shv extended-spectrum *β*-lactamases: neglected yet ubiquitous. Front. Microbiol..

[64] Livermore DM (2008). Defining an extended-spectrum *β*-lactamase. Clin. Microbiol. Infect..

[65] Bennett KM (2007). Implementation of antibiotic rotation protocol improves antibiotic susceptibility profile in a surgical intensive care unit. J. Trauma - Inj., Infect. Crit. Care.

[66] Karam G, Chastre J, Wilcox MH, Vincent JL (2016). Antibiotic strategies in the era of multidrug resistance. Crit. Care.

[67] Stiffler MA (2020). Protein structure from experimental evolution. Cell Syst..

[68] Fantini M, Lisi S, De Los Rios P, Cattaneo A, Pastore A (2020). Protein structural information and evolutionary landscape by in vitro evolution. Mol. Biol. Evolut..

[69] Matos-Cruz V (2017). Molecular prerequisites for diminished cold sensitivity in ground squirrels and hamsters. Cell Rep..

[70] Bautista DM (2007). The menthol receptor trpm8 is the principal detector of environmental cold. Nature.

[71] Yin Y (2019). Structural basis of cooling agent and lipid sensing by the cold-activated trpm8 channel. Science.

[72] Kao M-R, Yu S-M, Ua T-H, Ho D (2021). Improvements of the productivity and saccharification efficiency of the cellulolytic *β*-glucosidase d2-bgl in pichia pastoris via directed evolution. Biotechnol. Biofuels.

[73] Poelwijk FJ, De Vos MGJ, Tans SJ (2011). Tradeoffs and optimality in the evolution of gene regulation. Cell.

[74] Meyer AJ, Segall-Shapiro TH, Glassey E, Zhang J, Voigt CA (2018). Escherichia coli “marionette” strains with 12 highly optimized small-molecule sensors. Nat. Chem. Biol..

[75] Ellefson JW, Ledbetter MP, Ellington AD (2018). Directed evolution of a synthetic phylogeny of programmable trp repressors. Nat. Chem. Biol..

[76] Collins CH, Leadbetter JR, Arnold FH (2006). Dual selection enhances the signaling specificity of a variant of the quorum-sensing transcriptional activator luxr. Nat. Biotechnol..

[77] Tang SY, Fazelinia H, Cirino PC (2008). Arac regulatory protein mutants with altered effector specificity. J. Am. Chem. Soc..

[78] Snoek T (2020). Evolution-guided engineering of small-molecule biosensors. Nucleic Acids Res..

[79] Gane, A. et al. Protnlm: Model-based natural language protein annotation. Preprint at https://storage.googleapis.com/brain-genomics-public/research/proteins/protnlm/uniprot_2022_04/protnlm_preprint_draft.pdf (2023).

[80] Törönen P, Medlar A, Holm L (2018). PANNZER2: a rapid functional annotation web server. Nucleic Acids Res..

[81] Falda M (2012). Argot2: A large scale function prediction tool relying on semantic similarity of weighted gene ontology terms. BMC Bioinforma..

[82] Cantalapiedra CP, Hernández-Plaza A, Letunic I, Bork P, Huerta-Cepas J (2021). eggNOG-mapper v2: functional annotation, orthology assignments, and domain prediction at the metagenomic scale. Mol. Biol. Evolut..

[83] Pillai AS (2020). Origin of complexity in haemoglobin evolution. Nature.

[84] Tsan-Yuk Lam T (2020). Identifying sars-cov-2-related coronaviruses in malayan pangolins. Nature.

[85] Hatcher EL (2017). Virus variation resource - improved response to emergent viral outbreaks. Nucleic Acids Res..

[86] Facco E, Pagnani A, Russo ET, Laio A (2019). The intrinsic dimension of protein sequence evolution. PLoS Computat. Biol..

[87] Radhakrishnan A, Belkin M, Uhler C (2020). Overparameterized neural networks implement associative memory. Proc. Natl Acad. Sci..

[88] Abadi, M. et al. TensorFlow: Large-scale machine learning on heterogeneous systems. Software available from tensorflow.org. 10.48550/arXiv.1603.04467 (2015).

[89] Figliuzzi M, Barrat-Charlaix P, Weigt M (2018). How pairwise coevolutionary models capture the collective residue variability in proteins?. Mol. Biol. Evolut..

[90] Trinquier J, Uguzzoni G, Pagnani A, Zamponi F, Weigt M (2021). Efficient generative modeling of protein sequences using simple autoregressive models. Nat. Commun..

[91] Price MN, Dehal PS, Arkin AP (2009). Fasttree: Computing large minimum evolution trees with profiles instead of a distance matrix. Mol. Biol. Evolut..

[92] Hunter JD (2007). Matplotlib: a 2d graphics environment. Comput. Sci. Eng..

[93] Ashburner M (2000). Gene ontology: tool for the unification of biology. Nat. Genet..

[94] Binns D (2009). QuickGO: a web-based tool for Gene Ontology searching. Bioinformatics.

[95] Ziegler, C. Martin, J. Sinner, C. & Morcos, F. “Data from: Latent generative landscapes as maps of functional diversity in protein sequence space”. *Dryad, Dataset*10.5061/dryad.51c59zwbn (2023). 10.1038/s41467-023-37958-z PMC10113739 37076519

[96] Bateman A (2021). Uniprot: the universal protein knowledgebase in 2021. Nucleic Acids Res..

[97] Ziegler, C. Martin, J. Sinner, C. & Morcos, F. “LGL-VAE: Latent Generative Landscape - Variational Autoencoder“ *Github*10.5281/zenodo.7779323 (2023).

