# Supplementary material for: Latent generative landscapes as maps of functional diversity in protein sequence space
Source: Nat Commun. 2023 Apr 19;14:2222. doi: 10.1038/s41467-023-37958-z (PMC10113739; doi:10.1038/s41467-023-37958-z)
Supplement: Supplementary file 1 — Supplementary Information [file 41467_2023_37958_MOESM1_ESM.pdf]

# Supplementary Information for: Latent Generative Landscapes as Maps of Functional Diversity in Protein Sequence Space

Cheyenne Ziegler[1*], Jonathan Martin[1*], Claude Sinner[1], and Faruck Morcos[1,2,3**]

[1]Department of Biological Sciences, University of Texas at Dallas, Richardson, TX 75080, USA
[2]Department of Bioengineering, University of Texas at Dallas, Richardson, TX 75080, USA
[3] Center for Systems Biology, University of Texas at Dallas, Richardson, TX 75080, USA
[*]These authors contributed equally to this work.
[**]Corresponding author: faruckm@utdallas.edu

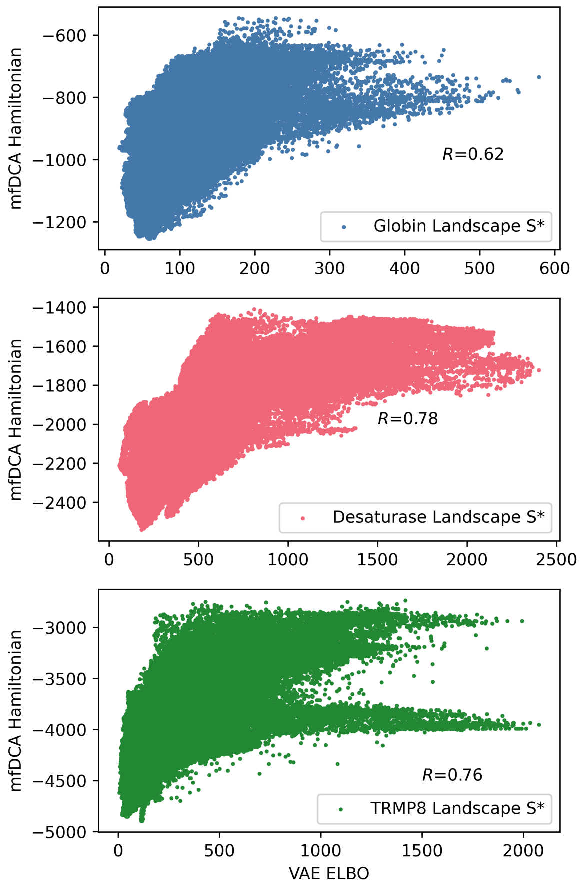


Supplementary Figure 1: Comparison of VAE and Hamiltonian scores. For each model listed, the max probability sequences generated for the Landscape are scored with the ELBO score from the VAE model which generated the landscape, and the Hamiltonian score from a mfDCA model fit with the same training sequences used to train the VAE.

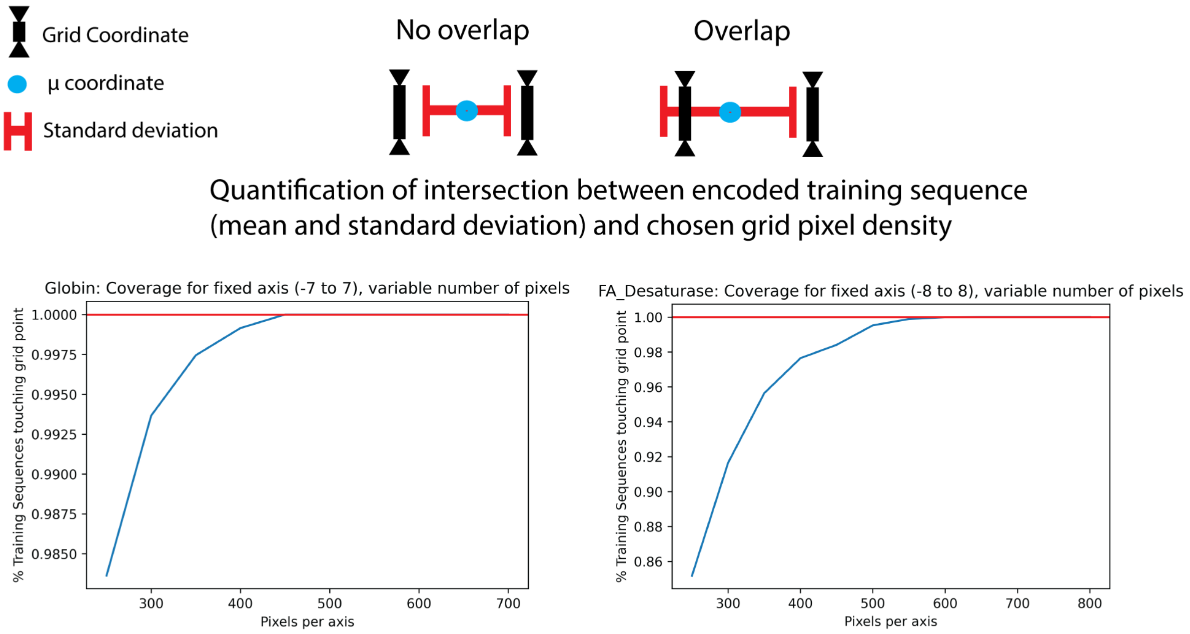


Supplementary Figure 2: Justification of LGL pixel density in relation to encoded training sequence standard deviations. For a range of pixel densities (for both axis) at a specific range (e.g. -7 to 7), the encoded mean and standard deviation of each training sequence was assessed against the entire grid. If either of the two dimensions for an encoded training sequence had some overlap with any grid coordinate, we consider it a success. Reported is the percentage of successes in the training set for the plotted pixel densities.

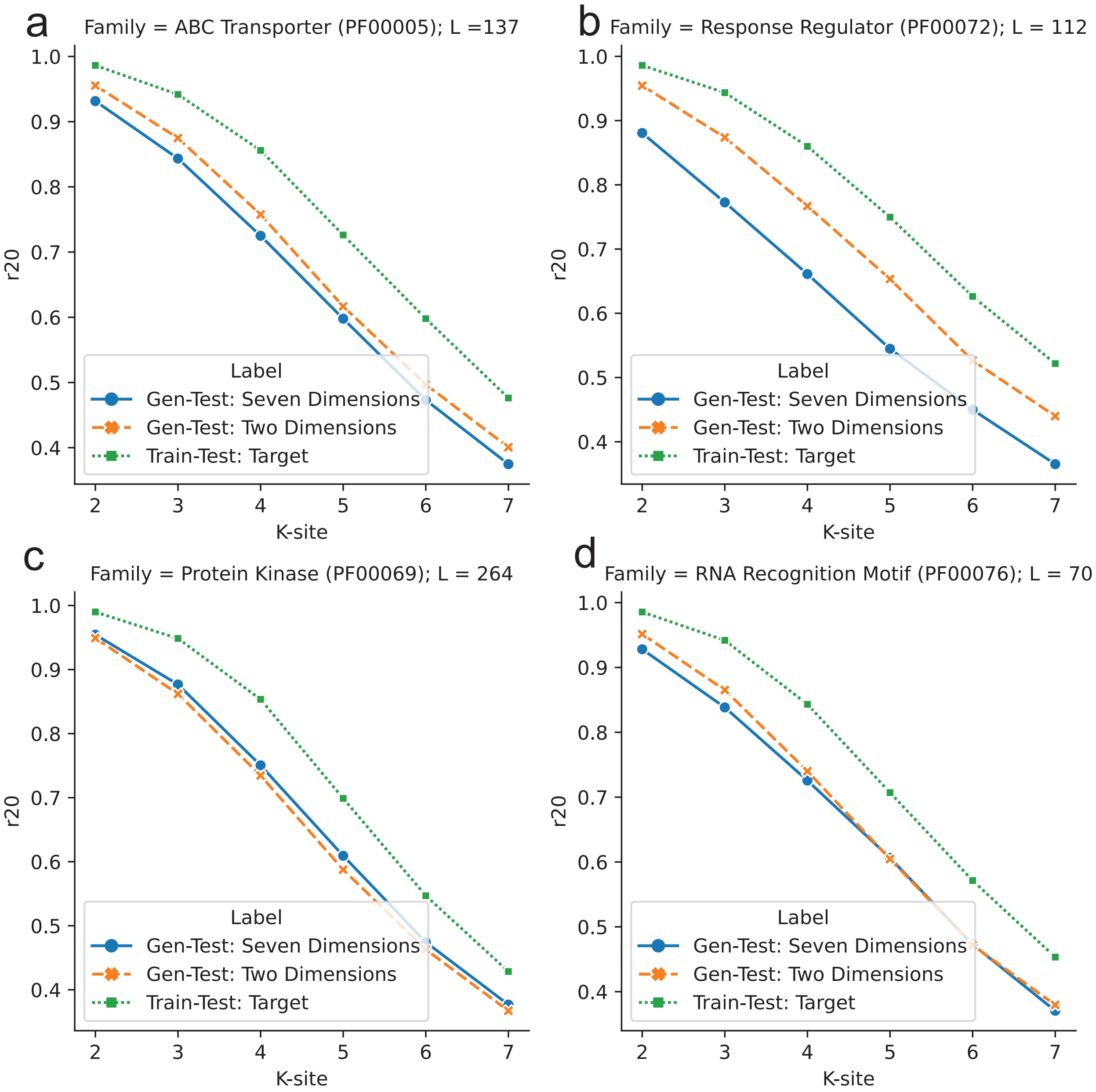


Supplementary Figure 3: r20 Scores for VAE trained with either two or seven dimensions over four families. VAE were trained on a training set, and generated sequences were compared to a test set. Train-Test compares the training set to the test set, and represents an estimated upper bound on the fit to the protein family's population statistics.

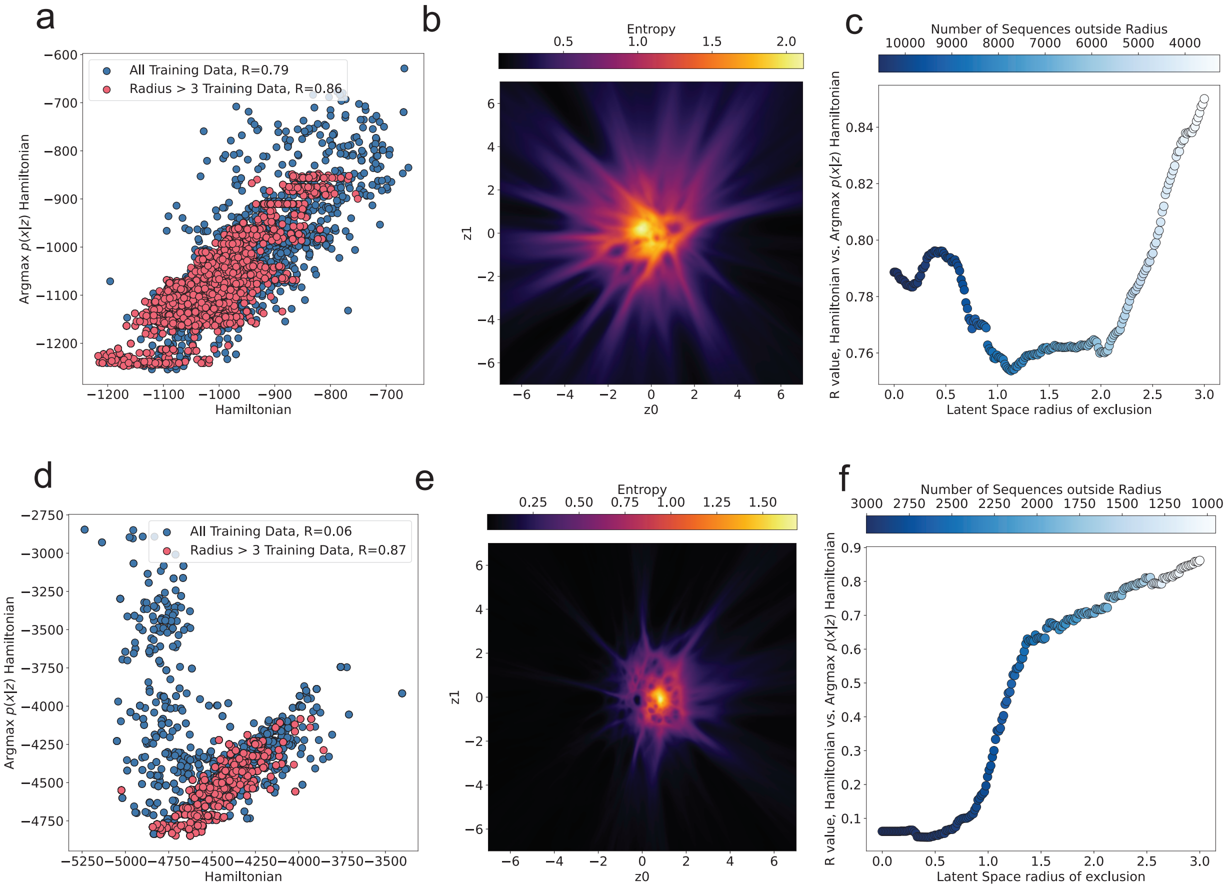


Supplementary Figure 4: Additional plots for Figure 2 for different families. (a-c) Globin family. (d-f) TRPM8 family.

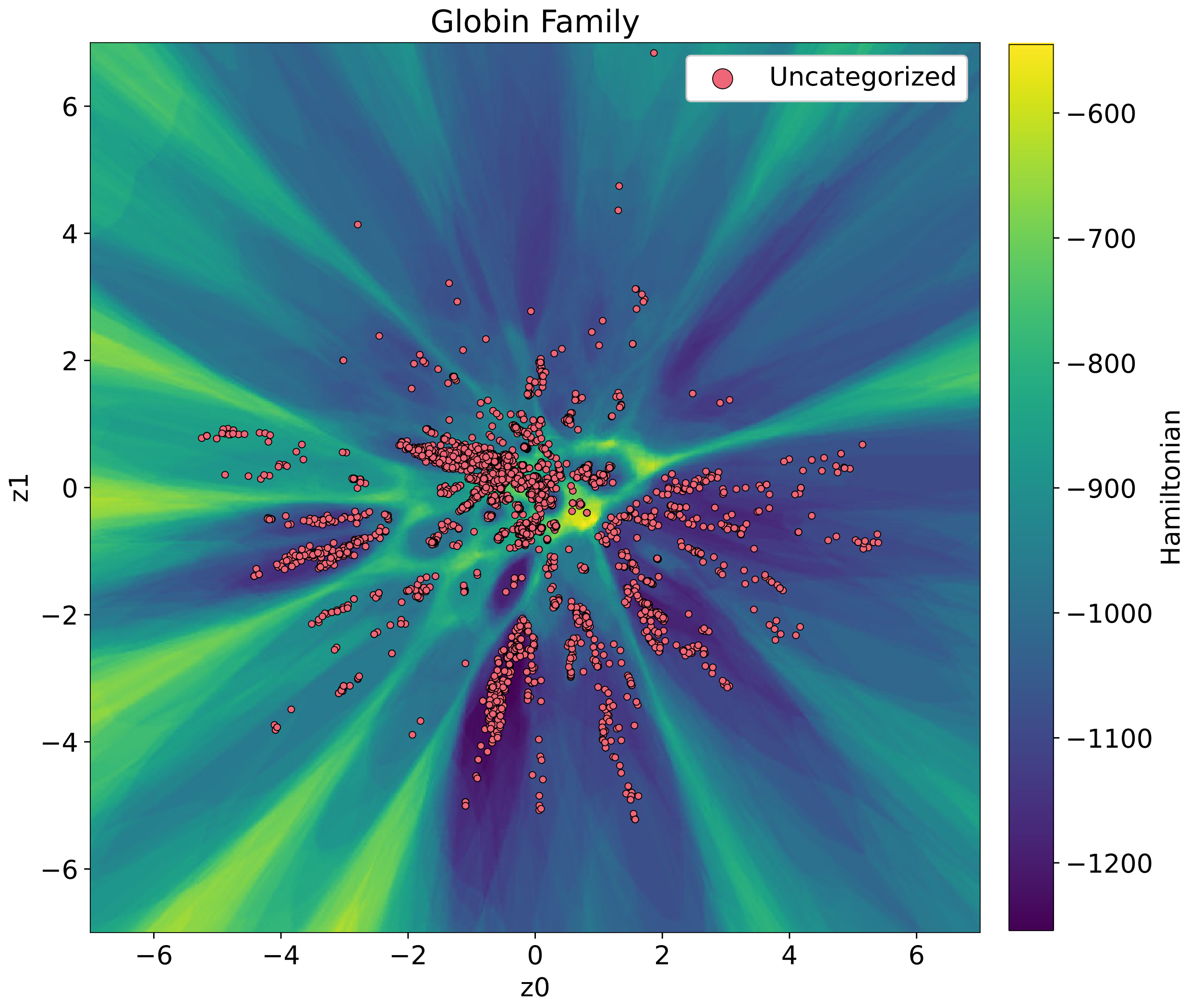


Supplementary Figure 5: Sequences from the Globin Pfam family which, when queried against Uniprot, had either no functional label (uncategorized) or a difficult to categorize function.

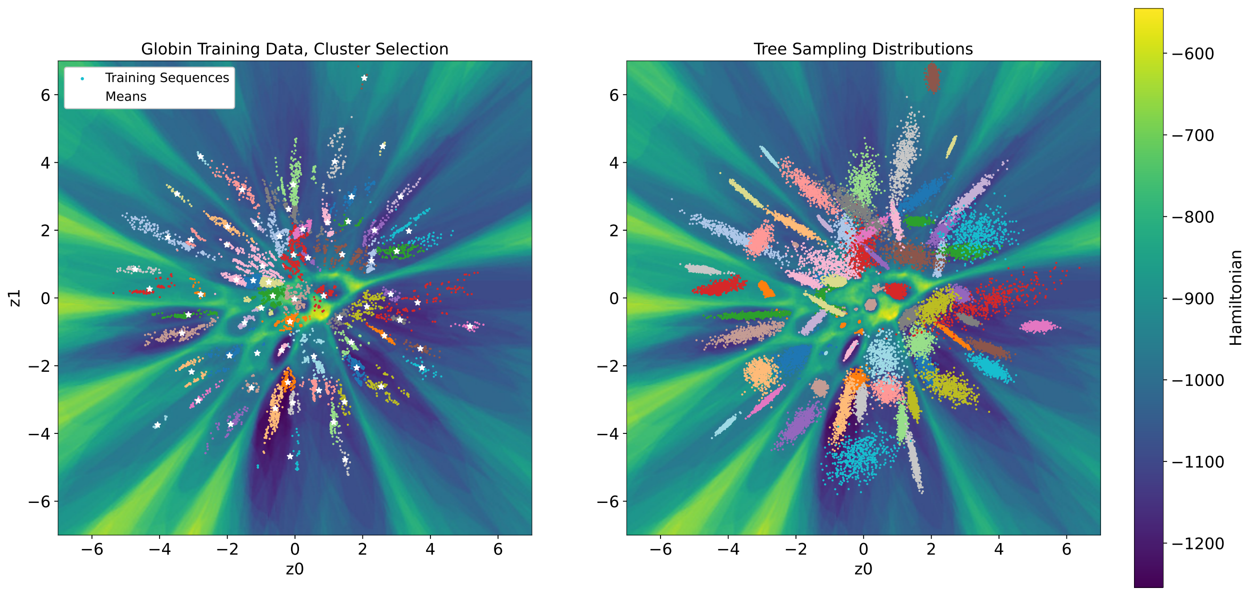


Supplementary Figure 6: Visual of tree sampling method. On the left, a Gaussian mixture model with 70 centers was fit to the training data after it was encoded into the Globin VAE model. Each cluster has a mean at the star point centered on each colored cluster, and each colored cluster is all of the sequences which belong to that cluster. On the right, the parameters for each of the 70 Gaussians were used to generate points, with each color corresponding to a different Gaussian distribution. In this way sequences were generated for tree comparison, where each real sequence and each synthetic sequence were identically labeled as being a member of their respective Gaussian.

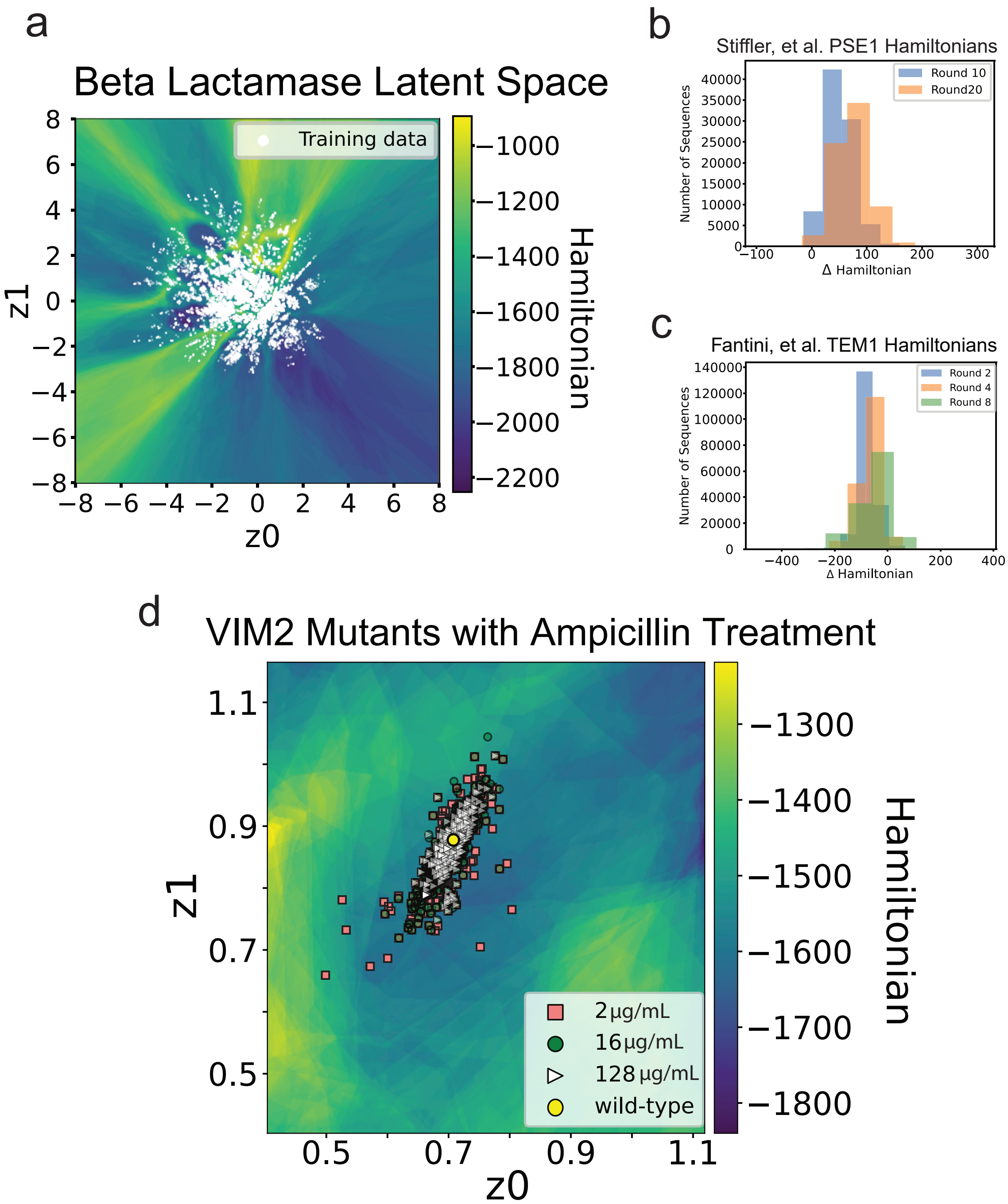


Supplementary Figure 7: Additional analyses from Stiffler et. al. and Fantini et. al. (a) Training sequences plotted onto the latent generative landscape for $\beta$-lactamase proteins.(b) $\Delta$ Hamiltonian between Stiffler, et al. $\beta$-lactamase mutants and wild-type PSE1. A negative $\Delta$ Hamiltonian indicates stronger fitness than wild-type PSE1, and a positive $\Delta$ Hamiltonian indicates weaker fitness than wild-type PSE1. (c) $\Delta$ Hamiltonian between Fantini, et. al. $\beta$-lactamase mutants and wild-type TEM1. A negative Hamiltonian indicates stronger fitness than wild-type TEM1, and a positive Hamiltonian indicates weaker fitness than wild-type TEM1. (d) VIM-2 neutral and positive fitness mutants as measured by growth under treatment with different concentrations of ampicillin as characterized by Chen, et al. LGL-VAE was trained on metallo-$\beta$-lactamases (PF00753). Results show how lowered selection pressure may increase the ability of mutants to occupy less favorable areas of the LGL.

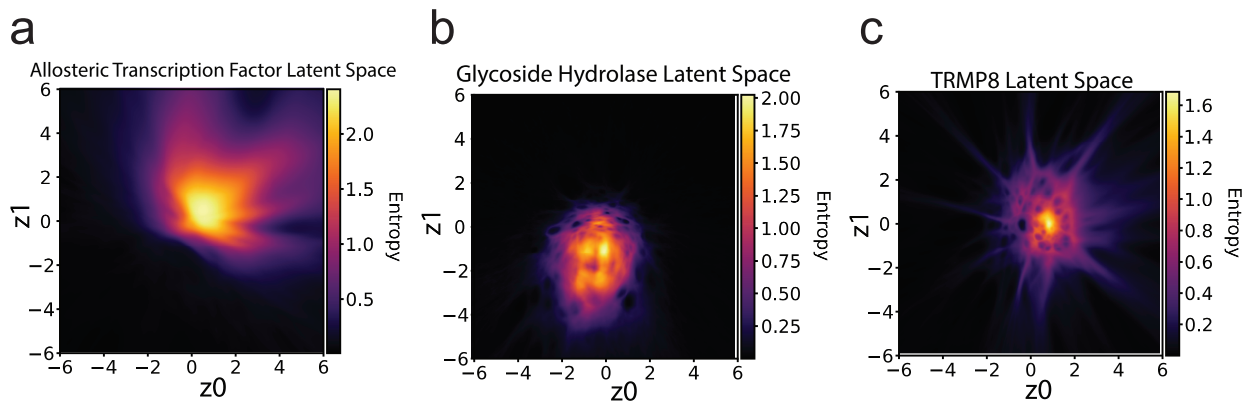


Supplementary Figure 8: Latent space by average entropy per amino acid position. All systems show high entropy towards center of plot. High entropy indicates that more unique sequences can be generated from the same latent space coordinates.

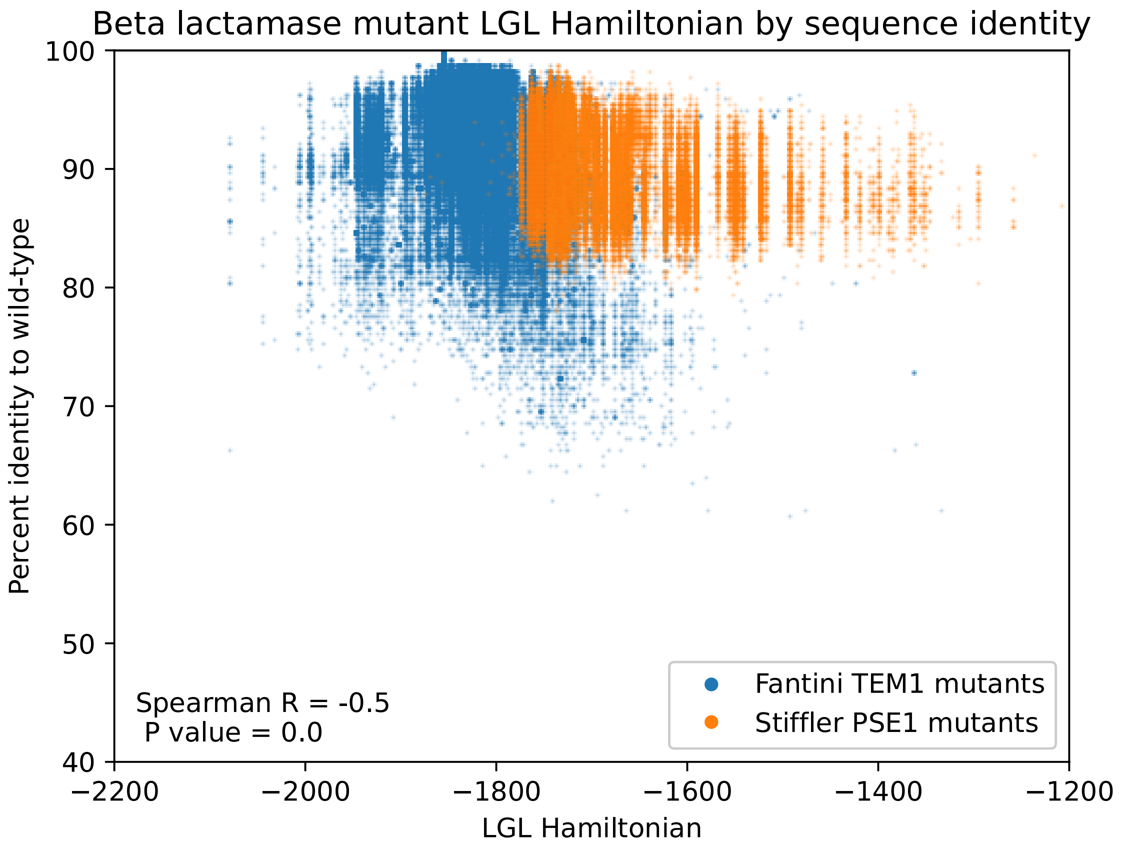


Supplementary Figure 9: $\beta$-lactamase sequence identity versus LGL Hamiltonian. While moderate to low correlation is expected, due to the nature of sequence statistics, the Pearson correlation of -0.5 (two-tailed) indicates that sequences given less favorable Hamiltonian in the LGL is not a consequence of distance to training data.

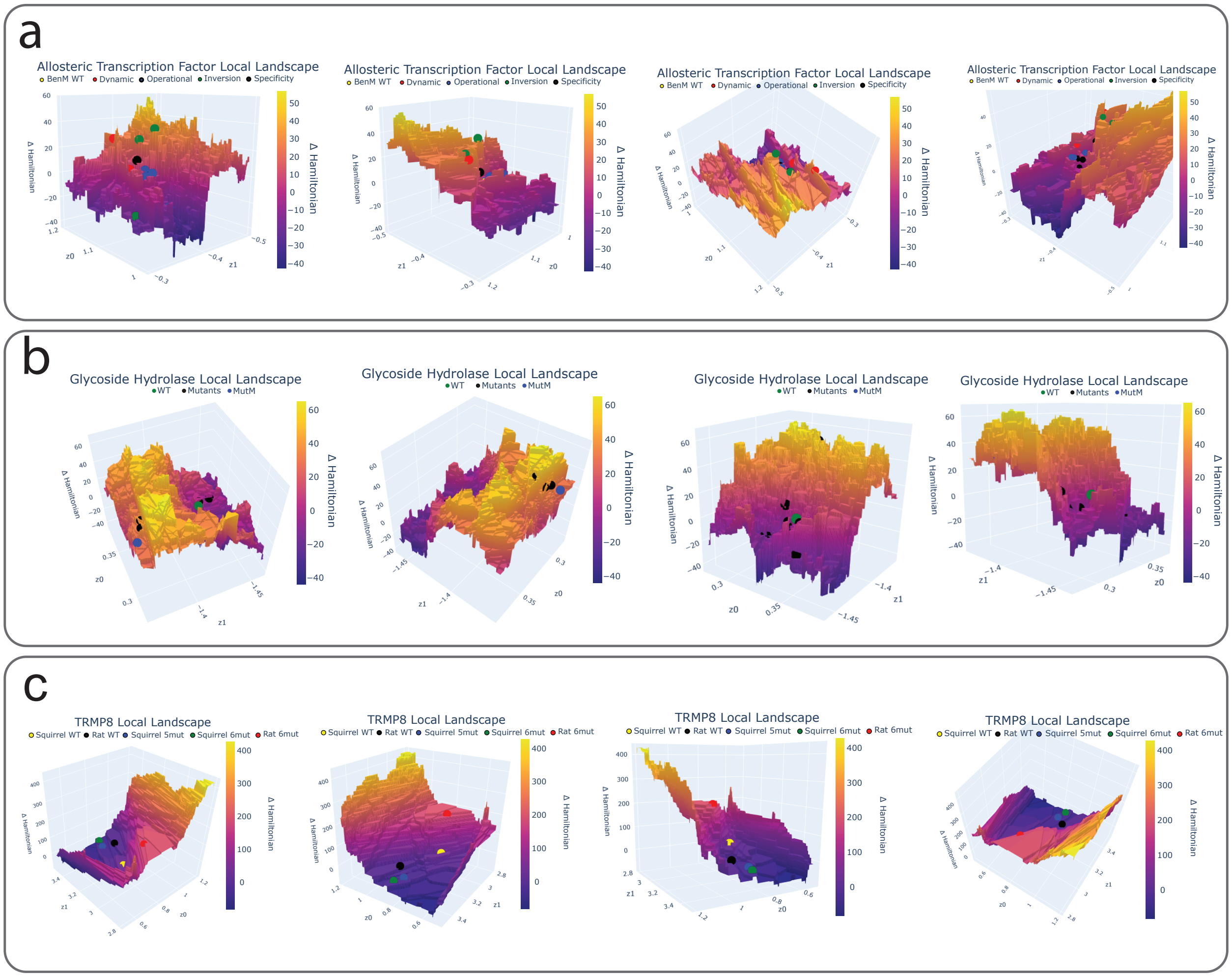


Supplementary Figure 10: 3-dimensional counter clockwise rotational plots: (a) Allosteric transcription factor latent generative landscape demonstrates movement of new sequences during guided-evolution through toggling selection pressures. (b) Glycoside hydrolase latent generative landscape shows how local barriers can divide areas of differing functions. (c) TRMP8 latent generative landscape shows increased function moving towards local basin.

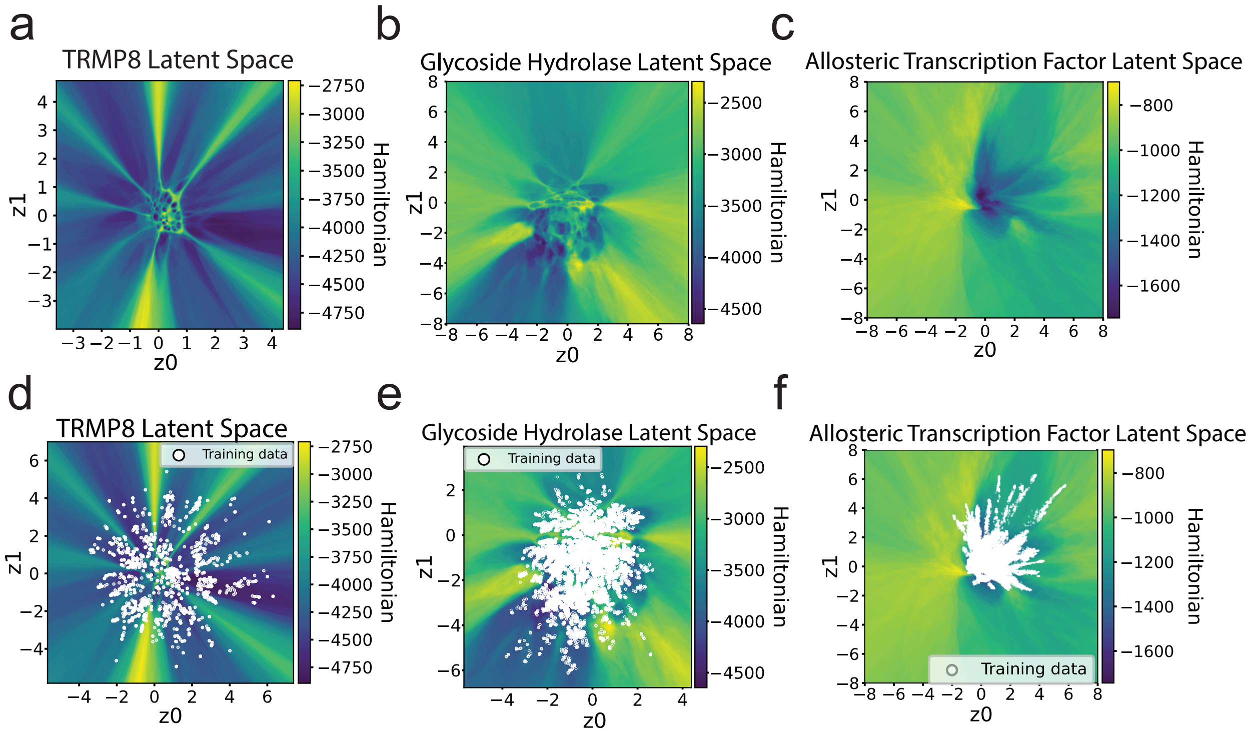


Supplementary Figure 11: Additional analyses of local latent space cases. (a) TRMP8 latent generative landscape. (b) Glycoside hydrolase latent generative landscape. (c) Allosteric transcription factor latent generative landscape. (d) TRMP8 latent generative landscape with training sequences. (e) Glycoside hydrolase latent generative landscape with training sequences. (f) Allosteric transcription factor latent generative landscape with training sequences.

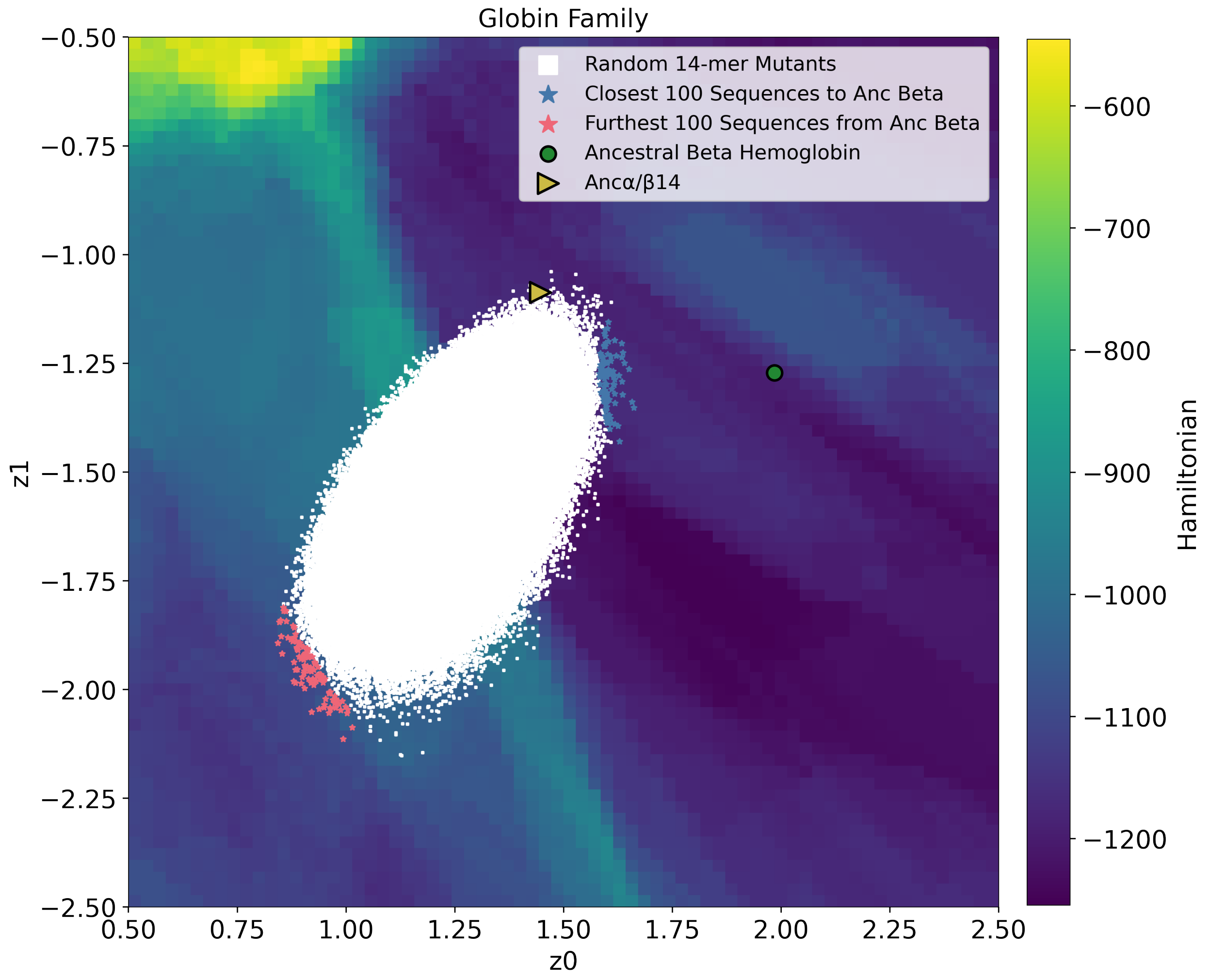


Supplementary Figure 12: Detail of random mutant selection from Figure 5a. The synthetic 14-mer sequences are plotted in the Globin VAE model, along with the ancestral beta hemoglobin sequence. The euclidean distance between the synthetic sequences and the ancestral sequence are compared, taking the closest 100 sequences and the furthest 100 sequences and collecting them into two separate multiple sequence alignments.

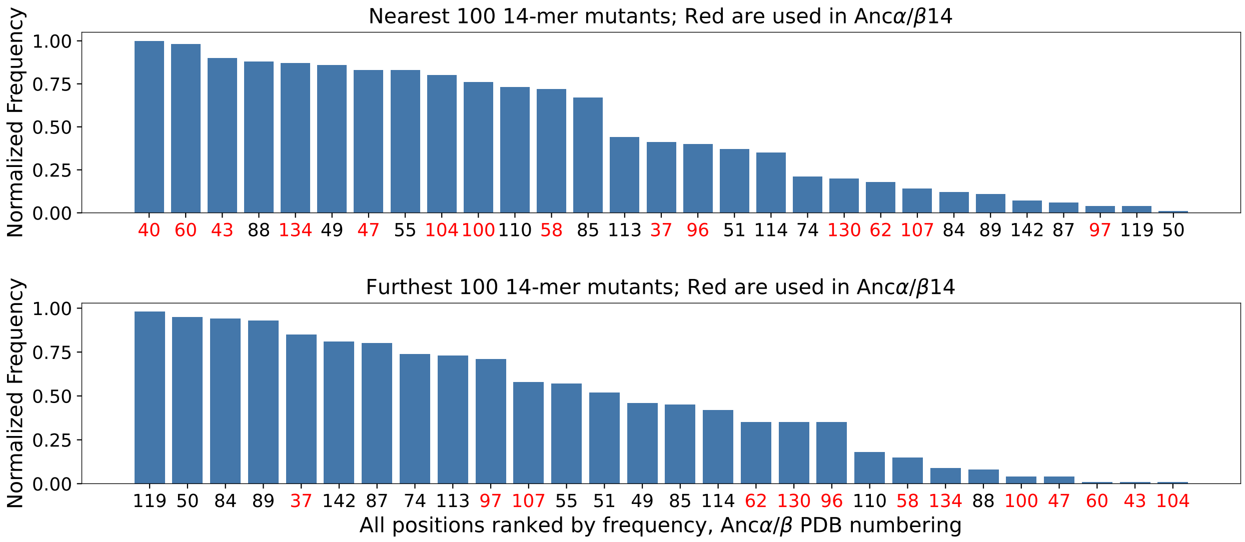


Supplementary Figure 13: From Figure 12, a histogram of which mutations are present in each group of collected sequences. Histogram is the normalized frequency of occurrence of mutant positions in each of the 100 14-mer mutants. Numbering is PDB coordinates from the Pillai et al. Datadryad deposition for Ancestral $\alpha$ / $\beta$ hemoglobin. Red positions are positions in the final 14-mer from the cited work.

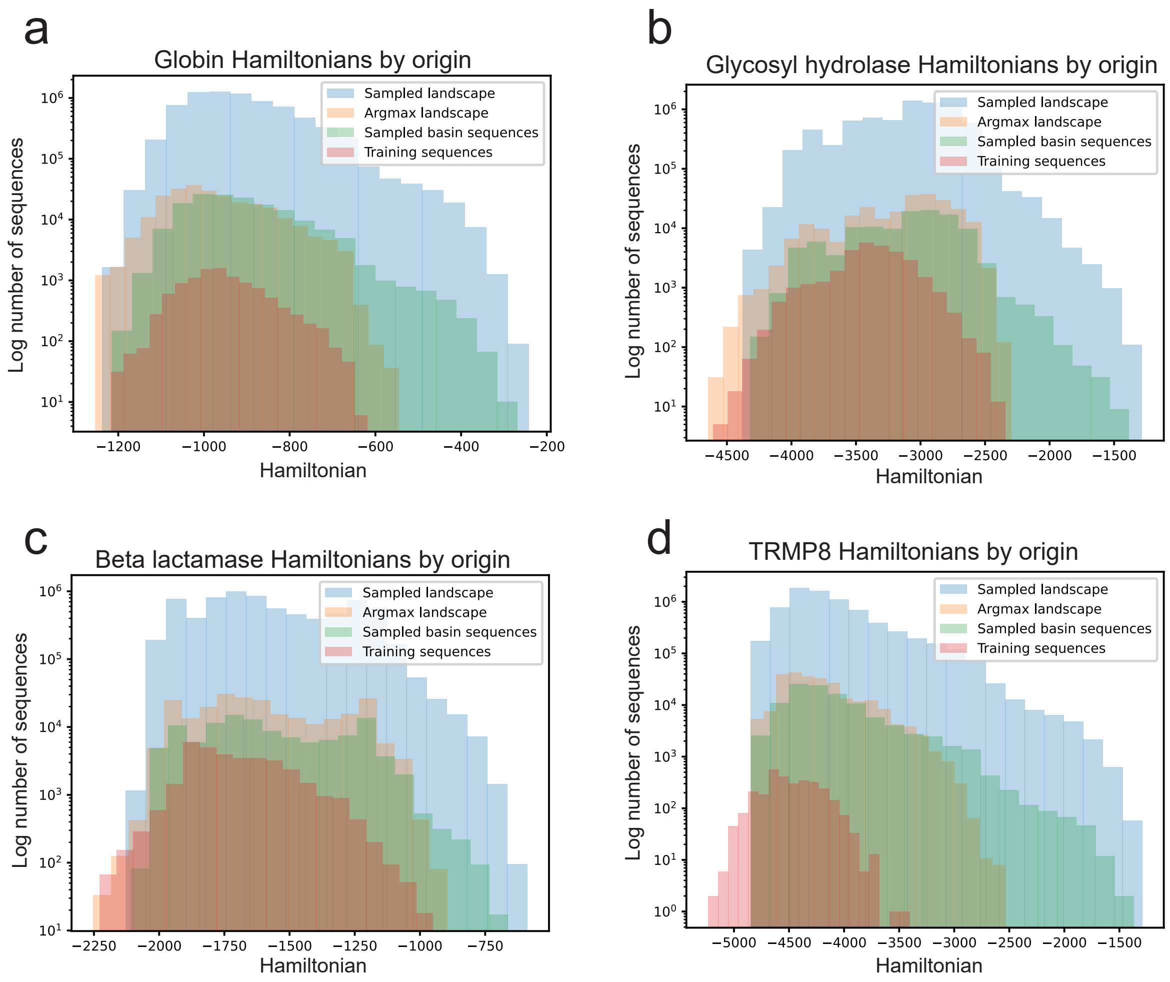


Supplementary Figure 14: Distribution of Hamiltonian for training sequences, argmax LGL, sampled LGL, and sampled LGL basins for (a) globin, (b) glycoside hydrolase, (c) $\beta$-lactamase, and (d) transmembrane protein 8 (TRMP8). Argmax distribution has closest fit to training sequence Hamiltonians. Sampled LGL Hamiltonians still overlap well with training sequence and argmax LGL, but exhibit shift towards more positive (less favorable) Hamiltonians. Sampled basins exhibit closer fit to natural sequences than the sampled landscape, further demonstrating how basins could be sampled to make sequences with higher fidelity to family statistics.

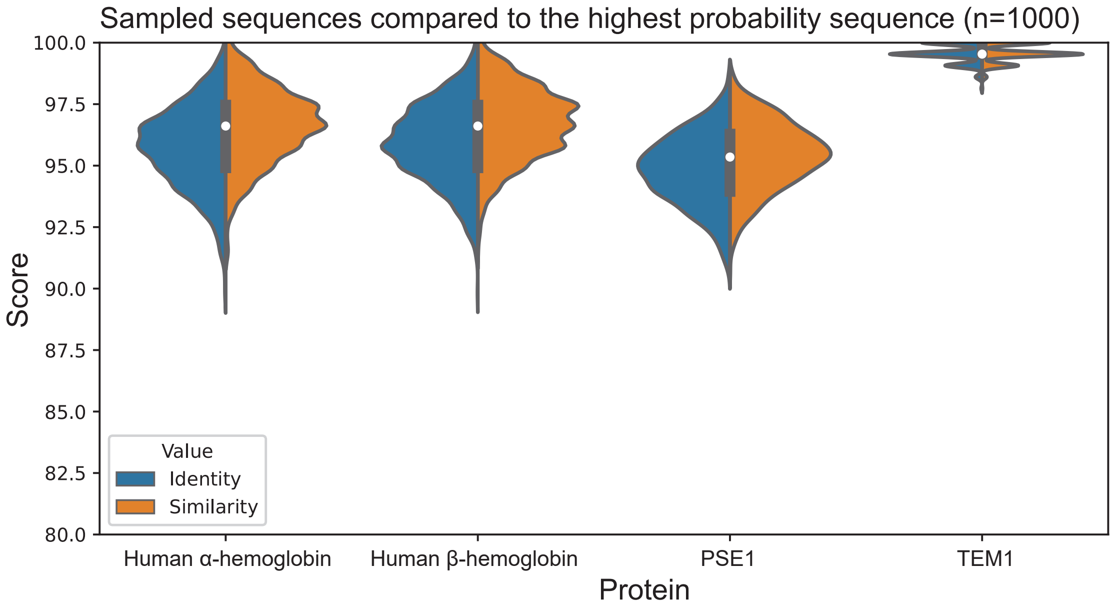


Supplementary Figure 15: Distribution of identity and similarity of sampled sequences compared to the maximum probability sequence for human $\alpha$-hemoglobin, human $\beta$-hemoglobin, PSE1, and TEM1. We see that the sampled sequences are 90% identical or more, with the average identity being 95% identical. For human $\alpha-$hemoglobin identity, $min$ = 89.3% is the bottom of the whisker, $max$ = 100% is the top of the whisker, $median$ = 95.76% is the white dot in the centre, $Q1$ = 94.92% is the bottom of the box, and $Q3$ = 96.61% is the top of the box. For human $\alpha-$hemoglobin similarity, $min$ = 91.25% is the bottom of the whisker, $max$ = 100% is the top of the whisker, $median$ = 96.61% is the white dot in the centre, $Q1$ = 95.76% is the bottom of the box, and $Q3$ = 97.46% is the top of the box. For human $\beta-$hemoglobin identity, $min$ = 89.83% is the bottom of the whisker, $max$ = 100% is the top of the whisker, $median$ = 95.76% is the white dot in the centre, $Q1$ = 94.92% is the bottom of the box, and $Q3$ = 97.46% is the top of the box. For human $\beta-$hemoglobin similarity, $min$ = 91.53% is the bottom of the whisker, $max$ = 100% is the top of the whisker, $median$ = 96.61% is the white dot in the centre, $Q1$ = 95.76% is the bottom of the box, and $Q3$ = 97.46% is the top of the box. For PSE1 identity, $min$ = 90.7% is the bottom of the whisker, $max$ = 98.6% is the top of the whisker, $median$ = 94.88% is the white dot in the centre, $Q1$ = 93.95% is the bottom of the box, and $Q3$ = 95.81% is the top of the box. For PSE1 similarity, $min$ = 90.7% is the bottom of the whisker, $max$ = 98.6% is the top of the whisker, $median$ = 95.35% is the the white dot in the centre, $Q1$ = 94.42% is the bottom of the box, and $Q3$ = 96.28% is the top of the box. For human TEM1 identity, $min$ = 98.14% is the bottom of the whisker, $max$ = 100% is the top of the whisker, $median$ = 99.53% is the white dot in the centre, $Q1$ = 99.53% is the bot, and $Q3$ = 100%. For human TEM1 similarity, $min$ = 98.14% is the bottom of the whisker, $max$ = 100% is the top of the whisker, $median$ = 99.53% is the white dot in the centre, $Q1$ = 99.53%, and $Q3$ = 100%. Similarity between sequences is not markedly higher than the identity, meaning that sequence sites exhibiting higher entropy are not distributed among two similar amino acids.

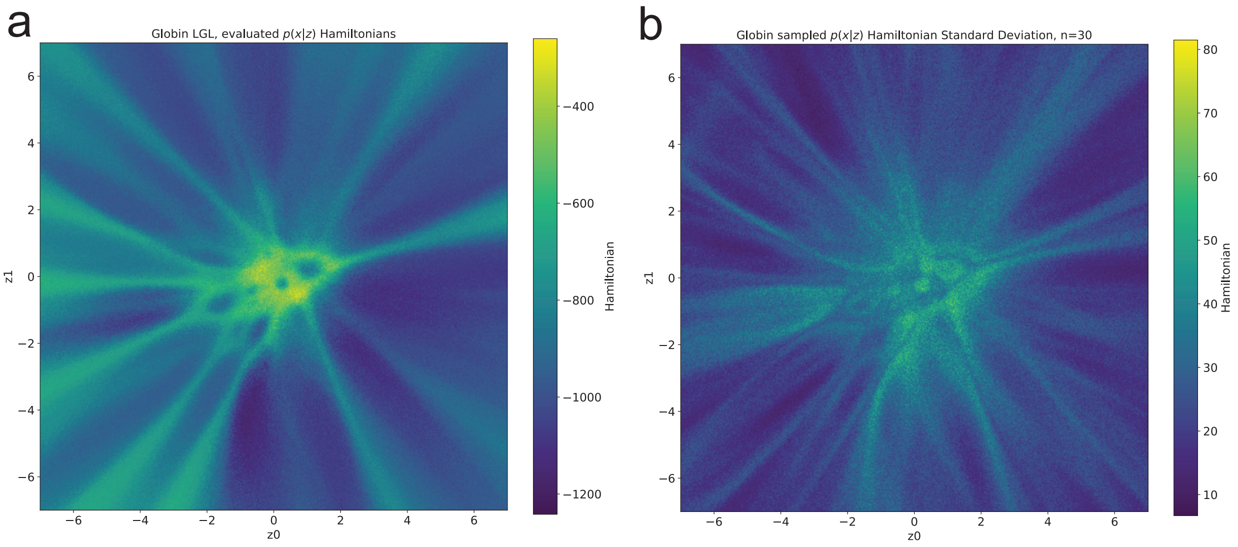


Supplementary Figure 16: Example of a sampled decoder landscape and the variance of the decoded Hamiltonian at each grid point. (a) For each grid point in the landscape, the distribution $p(x|\mathbf{z})$ is evaluated to generate a sequence, and that sequence is scored by the Hamiltonian. (b) At each grid point, 30 sequences are generated through evaluating $p(x|\mathbf{z})$, and the standard deviation of Hamiltonian values for these 30 sequences was computed and plotted.

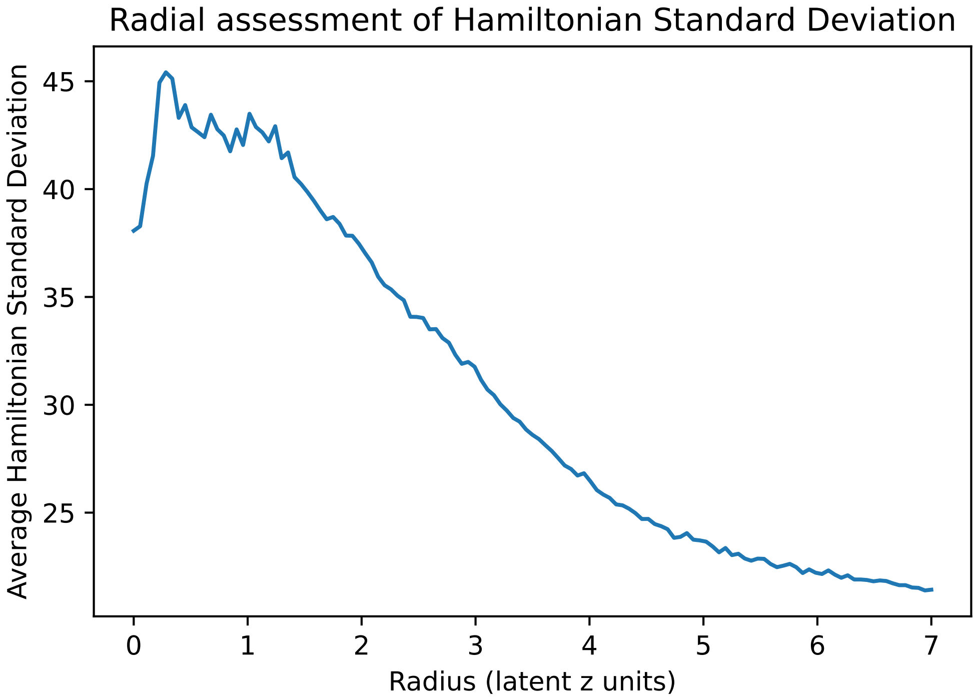


Supplementary Figure 17: Assessment of Hamiltonian standard deviation at grid coordinates as a function of the distance from the center. From accompanying Figure 16b, we create rings with a specified latent space radius, starting at the origin and expanding to the edge, and calculate the average Hamiltonian standard deviation for the pixels which lie on this ring at each radius.

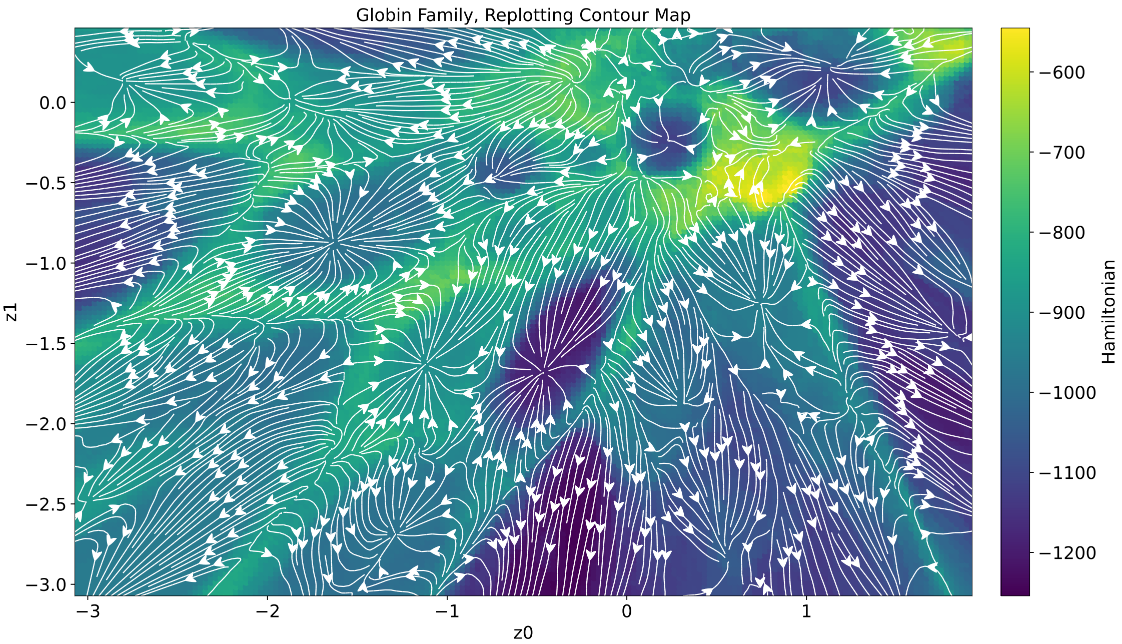


Supplementary Figure 18: Streamplot of vectors for re-encoded sequences, where the vector is the change in plotting coordinates from the input decoding coordinate to the encoded coordinate of the sequence generated with maximum probability.

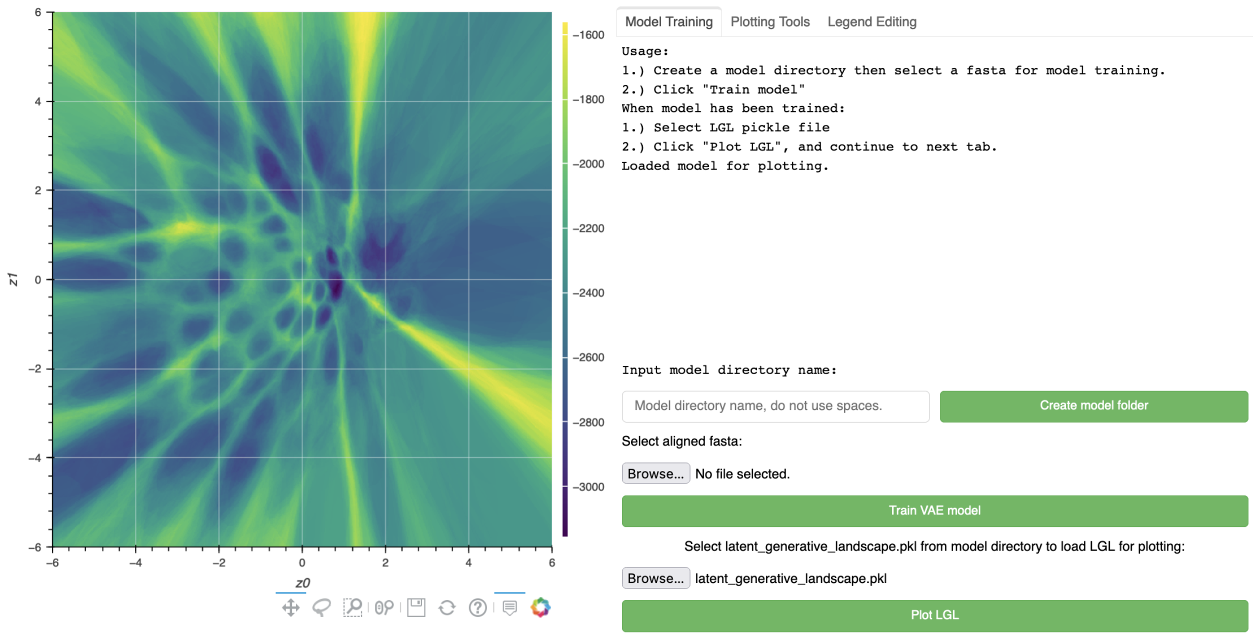


Supplementary Figure 19: Screenshot of LGL-VAE tool. Through this tool VAE models can be trained and sequences can be visualized within the landscape.

Supplementary Table 1: Ancestral sequence alignment used for mutant generation in Figure 6.

| Sequence ID | Sequence |
|---|---|
| QJC64256.1 ancestral alpha/beta hemoglobin | AEALARMFVTYPQTKTYFSHFSDAAEAIKGSPQVKAH |
| QJC64258.1 ancestral beta hemoglobin | AEALARMFVVYPWTKRYFSSFGNSAAAIMGNPKVKAH |
| | |
| QJC64256.1 ancestral alpha/beta hemoglobin | GKKVMGAIGEAVKHLDNLS---GALSKLSEKHAHKLR |
| QJC64258.1 ancestral beta hemoglobin | GKKVMGALGEAVKHLDNIK---ATFAKLSEKHSEKLH |
| | |
| QJC64256.1 ancestral alpha/beta hemoglobin | VDPHNFKLLSDCILVVLAVHFGADFTPEVHAAWDKFL |
| QJC64258.1 ancestral beta hemoglobin | VDPENFRLLGDCLIVVLAAHFGADFTPEVQAAWQKFL |
| | |
| QJC64256.1 ancestral alpha/beta hemoglobin | AVVATAL |
| QJC64258.1 ancestral beta hemoglobin | AVVASAL |

Supplementary Table 2: Multiple sequence alignments (MSAs) used as training data for each family, method of generation, and number of sequences in each alignment.

| Training MSAs | | |
|---|---|---|
| Family | HMMER Seed | Number of Sequences |
| Globin | PF00042 | 10,570 |
| Globin (ProtNLM) | PF00042 | 10,218 |
| FA_desaturase | PF00487 | 26,054 |
| Beta-lactamase2 | PF13354 | 34,088 |
| Metallo-beta-lactamase | PF00753 | 291,224 |
| Acetyltransferase | PF00583 | 382,278 |
| TRMP8 | Seed sequence used | 3,002 |
| Glycoside Hydrolase | Seed sequence used | 80,914 |
| Glycoside Hydrolase (ProtNLM) | Seed sequence used | 27,164 |
| TF Sensory Domain (LysR substrate) | PF03466 | 783,771 |
| Coronavirus Spike | Seed sequences used | 37,532 |
| tRNA Synthetase (ProtNLM) | PF00152 | 98,214 |
