# Supplementary material for: Latent generative landscapes as maps of functional diversity in protein sequence space
Source: Nat Commun. 2023 Apr 19;14:2222. doi: 10.1038/s41467-023-37958-z (PMC10113739; doi:10.1038/s41467-023-37958-z)
Supplement: Supplementary file 2 — Description of Additional Supplementary Files [file 41467_2023_37958_MOESM2_ESM.pdf]

<u>Description of Additional Supplementary Files</u>

Supplementary Video 1: Video was generated by plotting sequentially sequences by their deposition date. Unique sequences were clustered by their deposition date, and all sequences for a given date are plotted at the same time.
