# Supplementary material for: Latent generative landscapes as maps of functional diversity in protein sequence space
Source: Nat Commun. 2023 Apr 19;14:2222. doi: 10.1038/s41467-023-37958-z (PMC10113739; doi:10.1038/s41467-023-37958-z)
Supplement: Supplementary file 4 — Reporting Summary [file 41467_2023_37958_MOESM4_ESM.pdf]

nature portfolio

Corresponding author(s): Faruck Morcos

Last updated by author(s): Mar 29, 2023

Reporting Summary

Nature Portfolio wishes to improve the reproducibility of the work that we publish. This form provides structure for consistency and transparency in reporting. For further information on Nature Portfolio policies, see our Editorial Policies and the Editorial Policy Checklist.

Statistics

For all statistical analyses, confirm that the following items are present in the figure legend, table legend, main text, or Methods section.

n/a	Confirmed	
☐	☒	The exact sample size (*n*) for each experimental group/condition, given as a discrete number and unit of measurement
☐	☒	A statement on whether measurements were taken from distinct samples or whether the same sample was measured repeatedly
☐	☒	The statistical test(s) used AND whether they are one- or two-sided<br>*Only common tests should be described solely by name; describe more complex techniques in the Methods section.*
☐	☒	A description of all covariates tested
☐	☒	A description of any assumptions or corrections, such as tests of normality and adjustment for multiple comparisons
☐	☒	A full description of the statistical parameters including central tendency (e.g. means) or other basic estimates (e.g. regression coefficient) AND variation (e.g. standard deviation) or associated estimates of uncertainty (e.g. confidence intervals)
☐	☒	For null hypothesis testing, the test statistic (e.g. *F*, *t*, *r*) with confidence intervals, effect sizes, degrees of freedom and *P* value noted<br>*Give P values as exact values whenever suitable.*
☒	☐	For Bayesian analysis, information on the choice of priors and Markov chain Monte Carlo settings
☐	☒	For hierarchical and complex designs, identification of the appropriate level for tests and full reporting of outcomes
☐	☒	Estimates of effect sizes (e.g. Cohen's *d*, Pearson's *r*), indicating how they were calculated

Our web collection on statistics for biologists contains articles on many of the points above.

Software and code

Policy information about availability of computer code

Data collection	HMM seeds from PFAM families or a provided seed sequence (in Datadryad) were used to create multiple sequence alignments using hmmsearch (HMMER v3.3.2) against Uniprot databases (Swiss-Prot and TrEMBL UniProt Release 2021_03). GO annotations were retrieved using QuickGO API as provided by EMBL-EPI (https://www.ebi.ac.uk/QuickGO/api/index.html). FastTree version 2.1.11 was utilized. R package TreeDist was version 2.6.0. Tensorflow version 2.8.0 was used. ProtNLM software does not have releases or versions but can be found at https://github.com/google-research/google-research/tree/master/protnlm.
Data analysis	All code used to generate results is included in https://github.com/morcoslab/LGL-VAE with dependency versions located in requirements.txt. Landscape generation and plotting can be done using the interactive tool by following the instructions on the README. HPC scripts are included in the scripts folder and have a description of required inputs.

For manuscripts utilizing custom algorithms or software that are central to the research but not yet described in published literature, software must be made available to editors and reviewers. We strongly encourage code deposition in a community repository (e.g. GitHub). See the Nature Portfolio guidelines for submitting code & software for further information.

Data

Policy information about availability of data

All manuscripts must include a data availability statement. This statement should provide the following information, where applicable:

- Accession codes, unique identifiers, or web links for publicly available datasets
- A description of any restrictions on data availability
- For clinical datasets or third party data, please ensure that the statement adheres to our policy

The sequence, model, and validation data generated in this study have been deposited in the DataDryad database under accession code doi:10.5061/dryad.51c59zwbn [https://doi.org/10.5061/dryad.51c59zwbn]. The processed HMM seed data are available at PFAM on InterPro [https://www.ebi.ac.uk/interpro/] . The unprocessed sequence data are available at Swiss-Prot and TREMBL on Uniprot [https://www.uniprot.org/]. The plotting data generated in this study are provided in the Source Data file are available in the DataDryad database.

Human research participants

Policy information about studies involving human research participants and Sex and Gender in Research.

Reporting on sex and gender	N/A
Population characteristics	N/A
Recruitment	N/A
Ethics oversight	N/A

Note that full information on the approval of the study protocol must also be provided in the manuscript.

Field-specific reporting

Please select the one below that is the best fit for your research. If you are not sure, read the appropriate sections before making your selection.

☒ Life sciences ☐ Behavioural & social sciences ☐ Ecological, evolutionary & environmental sciences

For a reference copy of the document with all sections, see nature.com/documents/nr-reporting-summary-flat.pdf

Life sciences study design

All studies must disclose on these points even when the disclosure is negative.

Sample size	Sample sizes for training data was the maximum number of aligned sequences that pass preprocessing requirements. Sampling to generate sequences from latent space used the most probable sequence from the generated distribution of amino acid possibilities at each position.
Data exclusions	Sequences with over 20% contiguous gaps were excluded from the LGL-VAE training. This is because overabundance of gaps can negatively impact DCA statistical parameters and VAE learned parameters.
Replication	LGL-VAE parameters were chosen to increase replication stability. While each model presented has stable findings between at least 3 runs, there is always rotational variation and also variation between runs caused by the addition of noise that occurs in the VAE training process. LGL-VAE maps should be analyzed by considering relational differences between embedded sequences and the Hamiltonian scoring of sequence space around them.
Randomization	Randomization was implemented in LGL-VAE training by shuffling input sequences.
Blinding	Blinding was not necessary as all samples passing preprocessing were used and there was no manual selection of data.

Reporting for specific materials, systems and methods

We require information from authors about some types of materials, experimental systems and methods used in many studies. Here, indicate whether each material, system or method listed is relevant to your study. If you are not sure if a list item applies to your research, read the appropriate section before selecting a response.

Materials & experimental systems

n/a	Involved in the study
☒	☐ Antibodies
☒	☐ Eukaryotic cell lines
☒	☐ Palaeontology and archaeology
☒	☐ Animals and other organisms
☒	☐ Clinical data
☒	☐ Dual use research of concern

Methods

n/a	Involved in the study
☒	☐ ChIP-seq
☒	☐ Flow cytometry
☒	☐ MRI-based neuroimaging
